# Identification of genes influencing dendrite morphogenesis in developing peripheral sensory and central motor neurons

**DOI:** 10.1186/1749-8104-3-16

**Published:** 2008-07-10

**Authors:** Yimiao Ou, Barbara Chwalla, Matthias Landgraf, Donald J van Meyel

**Affiliations:** 1 Centre for Research in Neuroscience, McGill University, Cedar Ave, Montreal, QC, H3G 1A4, Canada; 2 Department of Neurology and Neurosurgery, McGill University, Montreal, QC, H3G 1A4, Canada; 3 McGill University Health Centre Research Institute, Montreal, QC, H3G 1A4, Canada; 4 Department of Zoology, University of Cambridge, Downing Street, Cambridge CB2 3EJ, UK

## Abstract

**Background:**

Developing neurons form dendritic trees with cell type-specific patterns of growth, branching and targeting. Dendrites of *Drosophila *peripheral sensory neurons have emerged as a premier genetic model, though the molecular mechanisms that underlie and regulate their morphogenesis remain incompletely understood. Still less is known about this process in central neurons and the extent to which central and peripheral dendrites share common organisational principles and molecular features. To address these issues, we have carried out two comparable gain-of-function screens for genes that influence dendrite morphologies in peripheral dendritic arborisation (da) neurons and central RP2 motor neurons.

**Results:**

We found 35 unique loci that influenced da neuron dendrites, including five previously shown as required for da dendrite patterning. Several phenotypes were class-specific and many resembled those of known mutants, suggesting that genes identified in this study may converge with and extend known molecular pathways for dendrite development in da neurons. The second screen used a novel technique for cell-autonomous gene misexpression in RP2 motor neurons. We found 51 unique loci affecting RP2 dendrite morphology, 84% expressed in the central nervous system. The phenotypic classes from both screens demonstrate that gene misexpression can affect specific aspects of dendritic development, such as growth, branching and targeting. We demonstrate that these processes are genetically separable. Targeting phenotypes were specific to the RP2 screen, and we propose that dendrites in the central nervous system are targeted to territories defined by Cartesian co-ordinates along the antero-posterior and the medio-lateral axes of the central neuropile. Comparisons between the screens suggest that the dendrites of peripheral da and central RP2 neurons are shaped by regulatory programs that only partially overlap. We focused on one common candidate pathway controlled by the ecdysone receptor, and found that it promotes branching and growth of developing da neuron dendrites, but a role in RP2 dendrite development during embryonic and early larval stages was not apparent.

**Conclusion:**

We identified commonalities (for example, growth and branching) and distinctions (for example, targeting and ecdysone response) in the molecular and organizational framework that underlies dendrite development of peripheral and central neurons.

## Background

Dendrites are the primary sites for the reception of sensory and synaptic input to neurons. This input is influenced by the architecture of the dendritic tree [1,2] and by the targeting of dendrites into appropriate territories [3,4]. For example, the length and tufted architecture of dendrites in the auditory brainstem of birds and mammals influences the tuning of coincidence-detecting neurons to optimal stimulus frequencies [5]. In the vertebrate spinal cord, specific targeting of motor neuron dendrites correlates with the precise matching with their presynaptic sensory afferents [4].

A current challenge for developmental neurobiologists is to uncover the cellular and molecular mechanisms that underlie the growth, branching and targeting of dendrites. The fruitfly, *Drosophila melanogaster*, has proven to be an effective model system for applying genetics to this issue [6-9]. *Drosophila *has neurons that are uniquely identifiable, with reproducible dendrite morphologies as intricate and diverse as those of vertebrates [2]. Moreover, *Drosophila *dendrites are also thought to be homologous to those of vertebrate neurons [7]. Within the peripheral nervous system (PNS) of *Drosophila*, studies to date indicate that genetic programs regulate the outgrowth, size, branching pattern and orientation of dendritic arborisation (da) sensory neurons [2,6]. Although a range of molecules implicated in da dendrite development have been identified, including cytoskeleton-associated proteins, small GTPases, transmembrane proteins, transcription factors and translation regulators, our understanding of da neuron dendrite morphogenesis remains far from complete [6,10-15]. Still less is known about the development of dendrites in the *Drosophila *central nervous system (CNS) [16]. Dendrites in the CNS differ from PNS sensory neuron dendrites in that they are not specialised for the reception of particular external stimuli [17-20] but instead form connections with presynaptic terminals of other neurons, and develop in a highly complex environment, the central neuropile.

In view of these fundamental differences, it is important to ask whether dendrites of peripheral and central neurons are shaped by distinct or shared mechanisms. To this end, we have carried out two comparable gain-of-function genetic screens in *Drosophila *to identify genes influencing dendrite morphogenesis. We used GAL4 driver lines [21] that express in either the da sensory neurons or an identified central motor neuron, RP2, for which we designed a novel mosaic expression system [22]. We screened a well characterized collection of 141 lines that carry independent insertions of the Gene Search (GS) P element [23], a potent UAS-based vector that can direct the expression of genes flanking the site of insertion [24]. We identified genes in each screen that influenced dendritic architecture. Some were novel, some were previously characterized, and 39% of the genes were common to both screens. However, the phenotypes arising from the genes identified in these screens revealed that fundamental differences may exist in the way peripheral and central neurons grow, branch, and find their targets.

## Results

### A gain-of-function screen for genes that influence the morphologies of peripheral neuron dendrites

#### Screening in embryos

To identify genes affecting da dendrite morphogenesis, we focused on da neurons of the dorsal PNS cluster, visualised selectively by *GAL4*^109(2)80 ^and a transgene encoding a membrane-targeted green fluorescent protein (GFP) reporter, *UAS-mCD8::GFP *[15,21]. We crossed each of 141 GS expression lines into this background and assayed in late stage 17 *Drosophila *embryos the effects on: overall PNS integrity; the number and position of GFP-positive cell bodies in the dorsal cluster; the extent of dendrite outgrowth of dorsal cluster da neurons; and their pattern of branching. The results of this screen are reported in Table 1.

**Table 1:** Dendrite morphology phenotypes observed in da neurons

			Observed increases (+) or decreases (-)							
				
			Embryos (stage 17)		Larvae (third Instar)^a^					
					
								Class IV		
									
Line	Cytological location	Closest gene	Growth	Branching	Class I	Class II growth	Class III	Growth	Branching	Other observations
***shrub*-like effects**										
GSd034	3R;100D1	*ttk*	—		o	o	o	o	o	
GSd446	3R;100D1	*ttk*	—		o	o	o	o	o	
GSd468	3R;100D1	*ttk*	—		o	o	o	o	o	
GSd462^b^	3R;100D1 3R;92F1	*ttk Stat92E*	—		o	o	o	o	o	
										
***PcG*-like effects**										
GSd219	2R;57A6	*bl*							—	
GSd226	2L;21D1	*cbt*							—	
GSd220	2R;60A6	*ken*							—	
GSd247	2R;57A6	*mir-313*							—	
GSd472	2R;47D6	*shn*							—	
GSd469	3R;97E11	*woc*							—	
										
***cut*-like effects**										
GSd324	2L;32E2	*ab*				—	—	—	—	
GSd331	3R;98F13	*CG11897*				—	—		—	
GSd454	2L;30B5	*CG33298*				—	—	—	—	
GSd233	3R;88A4	*foxo*				—	—	—	—	
GSd411	3R;88A5	*foxo*				—	—	—	—	
GSd327	2L;38E3	*Hr38*				—	—	—	—	GFP intensity reduced
GSd500	3L;70D7	*stwl*					—		—	
										
**Other**										
GSd430	2R;59F1	*apt*					—		—	
GSd325	3R;92B3	*bnl*							+/—	
GSd332	3R;92F2	*bon*					—		—	
GSd321	3R;91F4	*CG11779*					—		—	GFP intensity reduced
GSd239	3R;86E11	*CG14709*					—		—	
GSd450	2L;38D5	*CG2617*			—	—	—	—	—	Fewer than eight multidendritic (md) neurons of dorsal cluster express GFP
GSd422	2R;42E1	*CG33558*		+	o	o	o	o	o	
GSd211	3R;87D7	*CG7518*			—	—	—	—	—	GFP expressing cell bodies and dendrites appear degenerative
GSd496	2L;29F8	*CG9582*		—	o	o	o	o	o	Similar to effect of activated cdc42
GSd066	3L;61B3	*E(bx)*							+/—	
GSd451	3L;61B3	*E(bx)*							+/—	
GSd492	3L;61B3	*E(bx)*							+/—	
GSd402	3R;99A1	EST:EN05557/EN06658			—	—	—	—	—	Fewer than eight md neurons express GFP
GSd214	2L;24A2	*for*					—		—	
GSd328	2L;24A4	*for*					—		—	
GSd312	3R;99E4	*hdc*					—		—	
GSd113	2L;26B5	*Kr-h1*				—		—	—	
GSd429	2R;47A12	*lola*					—		—	
GSd484	3R;93D9	*mod(mdg4)*			+		—		—	Similar to spineless mutations, but lacks effect on class II
GSd302	3R;91F4	*nos*					—		—	
GSd428	2L;36E3	*PFE*					—		—	
GSd236	3R;94E13	*pnt*					—		—	
GSd420	3R;94E13	*pnt*					—		—	
GSd458	2R;47A13	*psq*			+	—	—	—	—	Fewer than eight md neurons express GFP, reduced GFP intensity
GSd231	2R;52E5	*Rho1*	—	+	o	o	o	o	o	Reduced dendritic field, but terminals have exuberant branching
GSd431	2R;52E5	*Rho1*	—	+	o	o	o	o	o	Reduced dendritic field, but terminals have exuberant branching

^a^Boxes marked with an 'o' indicate that larval phenotypes were not assessed for those GS lines that had severe effects in embryos.

^b^Line GSd462 harbours two insertions of the GS element.

**Figure 1:**
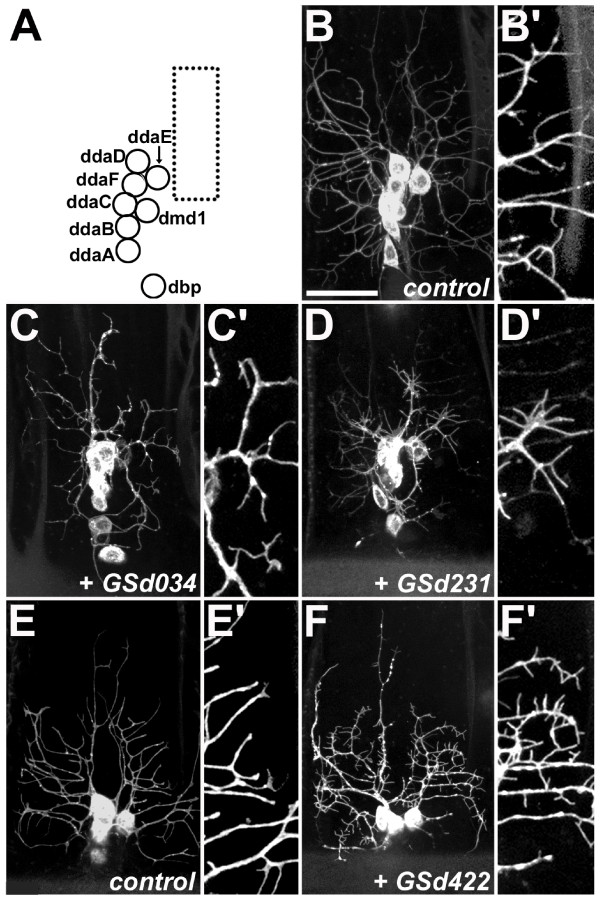
**Embryonic da dendrite screen – examples of phenotypes.(a) **Cartoon showing the relative positions of cell bodies of dorsal cluster multidendritic (md) neurons in late stage 17 embryos. The dotted box indicates the region of dendritic field chosen for enlargement in each of (b',c',d',e',f'). **(b,b') **In control animals, the dendritic trees of the six da neurons in the dorsal cluster can be visualized with *GAL4*^109(2)80 ^driving membrane-targeted GFP (*UAS-mCD8::GFP*). **(c,c') **Misexpression of GSd034: dilation of primary and reduced outgrowth of higher order dendritic branches. **(d,d') **Misexpression of GSd231: reduced dendritic field size with residual branching. **(e,e') **Slightly younger control animal, though still late stage 17, for comparison with (f,f'). **(f,f') **Misexpression of GSd422: production of filopodial spine-like protrusions. All images are maximal Z-projections of stacked confocal images. Anterior is left and ventral is down in all panels. Scale bar in (b) = 20 μm and applies to (c-f) also.

Our screen identified eight GS lines that, at embryonic stages, caused severely reduced dendrite outgrowth and/or branching, or increased branching (Figure 1). For example, misexpression of GSd034 resulted in thickened lower order dendrites and reduced outgrowth of higher order dendritic branches (Figure 1c). Misexpression of GSd231 led to reduced dendritic growth as reflected by reduced area of the dendritic field (Figure 1d), though profuse branching was retained at the terminals of the shortened primary branches. A third example is misexpression of GSd422, which caused the formation of ectopic spine-like protrusions from the main branches of all dorsal cluster da neurons (Figure 1f). These examples demonstrate that gene misexpression can modulate specific aspects of dendritic development, such as growth and branching, and that the regulation of these two processes is genetically separable.

#### Screening in larvae

**Figure 2:**
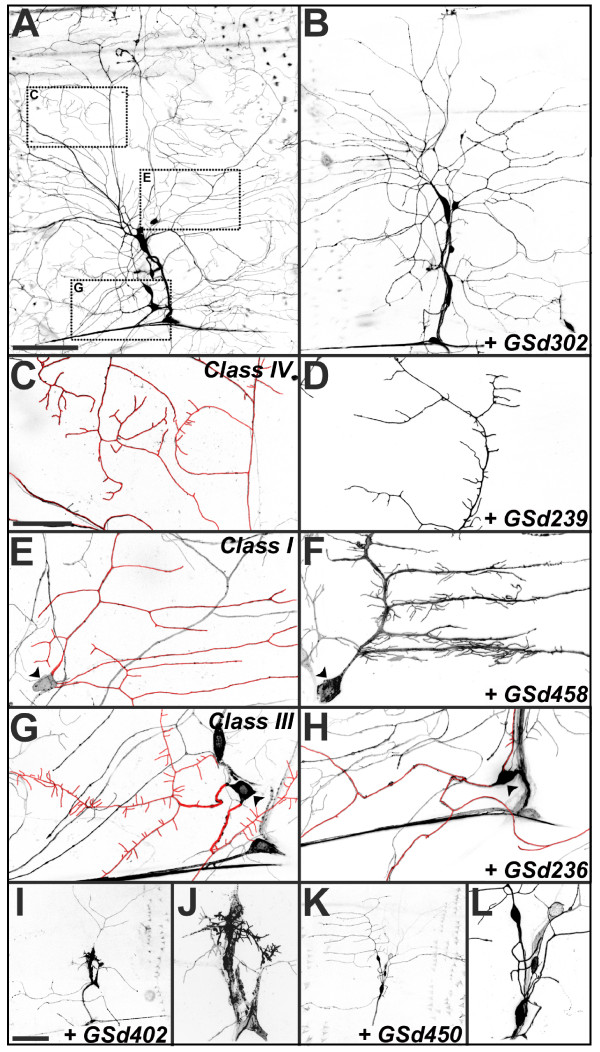
**Larval da dendrite screen – examples of phenotypes. (a) **In a control third instar larva (*GAL4*^109(2)80^, *UAS-mCD8::GFP/+*), one can visualize the eight multidendritic neuron cell bodies of the dorsal cluster and the fields occupied by da neuron dendrites. Dotted boxes indicate fields examined at higher power in (c,e,g) to illustrate regions occupied primarily by the dendrites of class IV (c) (ddaC), class I (e) (ddaE), and class III (g) (ddaA) da neurons. **(b) **Misexpression of GSd302: severe reduction of higher order branches in class III and class IV da neurons, though the growth of primary branches of these and other da neurons appears intact. **(c) **Region in control highlighting (in red) the higher order branches of the class IV ddaC neuron. **(d) **Misexpression of GSd239 reduced the length of higher order branches of ddaC, with no obvious reduction in branch number. **(e) **Control ddaE (arrowhead at cell body), a class I da neuron that ordinarily has a simple pattern of lower order dendrite branches (highlighted in red). **(f) **Misexpression of GSd458 caused numerous small branches to emerge from ddaE (arrowhead at cell body). **(h) **Compared to controls (as in (g)), misexpression of GSd236 severely reduced numbers of spine-like protrusions in the class III neuron ddaA (cell body marked with arrowhead, dendrites highlighted in red). **(i-k) **Misexpression GSd402 and GSd450 caused severe reduction of dendrite outgrowth and branching (i,k), often with fewer GFP-labelled da neurons and signs of neuronal degeneration (higher power in (j,l)). All images are maximal Z-projections of stacked confocal images. Anterior is left and ventral is down. Scale bar in (a) = 100 μm and applies to (b) also. Scale bar in (c) = 30 μm and applies to (d-h) also. Scale bar in (i) = 100 μm and applies to (k) also.

35 GS lines caused mild but reproducible defects at embryonic stages. We characterised their phenotypes at a later developmental stage, analyzing the same dorsal cluster da neurons in wandering third instar larvae (Figure 2). In larvae, four da neuron classes (I-IV) of increasing dendritic complexity and size can be readily observed, and at least one representative from each class resides in the dorsal cluster. We examined whether GS misexpression influenced dendritic morphology of the class I neurons ddaD and ddaE, the class II neuron ddaB, the class III neurons ddaA and ddaF, and the class IV neuron ddaC (Figure 2a). Class I da neurons have the simplest pattern, having relatively few primary dendrites with interstitial secondary and tertiary branches oriented in the anterior-posterior direction (Figure 2e). The dendrites of class II da neurons are long and typically have symmetric bifurcations. Class III dendrites are characterized by short spine-like protrusions emanating from long main branches (Figure 2g). Class IV dendrites show highly complex branching patterns that innervate large regions of the body wall (Figure 2c) [25].

**Figure 3:**
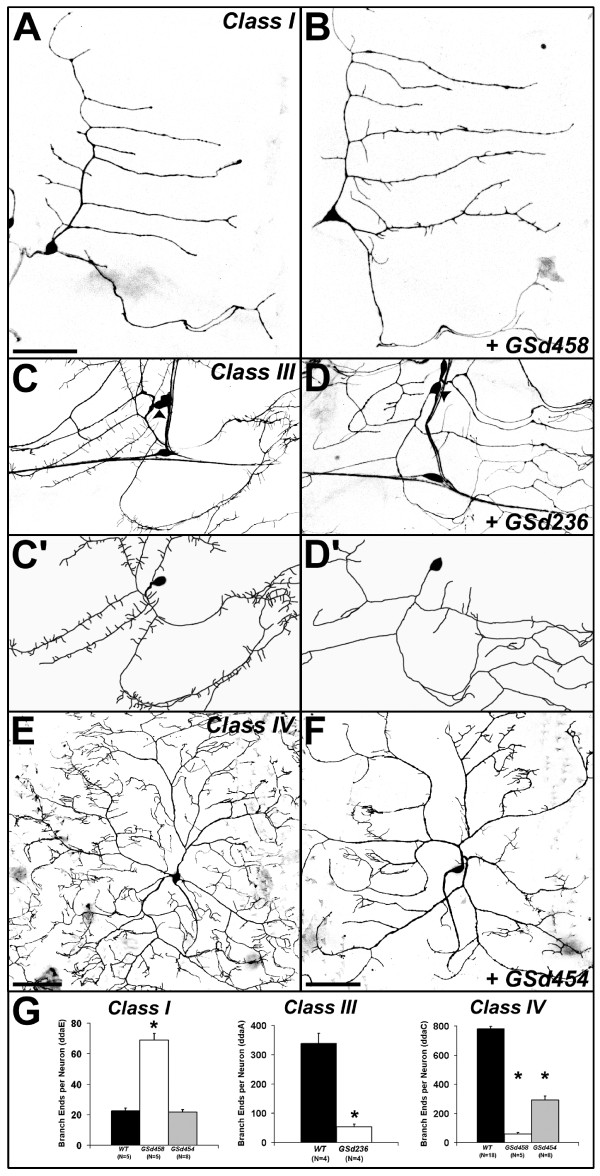
**GS misexpression with class-specific Gal4 drivers in larval da neurons.(a) **Control class I da neuron ddaE visualized with *GAL4*^221 ^driving *UAS-mCD8::GFP*. **(b) **Misexpression of GSd458 caused increased numbers of small dendritic branches, though the primary branches were unaffected. **(c) ***C161-GAL4 *drives expression in classes I-III, but not class IV, allowing better visualization of spine-like protrusions on the class III neuron ddaA. Arrowheads in (c,d) mark the cell body of ddaA. **(c') **Tracing of ddaA cell body and dendrites in (c). **(d) **Misexpression of GSd236: primary dendrites of ddaA are devoid of spine-like protrusions. **(d') **Tracing of ddaA cell body and dendrites in (d). **(e) ***ppk1.9-GAL4 *is a class IV da neuron driver, revealing the complex dendritic tree of ddaC. **(f) **Misexpression of Gsd454: reductions in the number and growth of higher order branches of ddaC. **(g) **Quantification of branch ends per neuron for the genotypes shown in (a-f), showing class specificity of branching defects. In class I ddaE neurons (left), GSd458 increases branching dramatically (asterisk denotes *t*-test, *P *< 1e^-5^), while GSd454 has no effect. In class IV ddaC neurons (right), both GSd458 and GSd454 reduce branching relative to controls (wild type (WT); asterisks denote *t*-tests, both *P *< 1e^-8^). In both cases, the total length of the dendritic arbor was dramatically reduced (control (WT) = 17,389 ± 422 μm versus GSd454 = 8,544 ± 657 μm (*t*-test *P *< 1e^-10^) or versus GSd458 = 2,650 ± 296 μm (*t*-test, *P *< 1e^-16^). Since higher order branches were reduced but the growth of primary dendrites was mostly unaffected, there was no effect on dendritic field area (for example, control = 304,899 ± 7,115 μm^2 ^versus GSd454 = 301,475 ± 9,141 μm^2^; *t*-test *P *> 0.8). In class III ddaA neurons (middle), GSd236 dramatically reduced the number of short spine-like protrusions (*t*-test, *P *< 0.003), but had no effect on the total length of primary dendrites (control = 1,736 ± 137 μm versus GSd236 = 2,132 ± 157 μm; *t*-test *P *> 0.1). All images are maximal Z-projections of stacked confocal images. Anterior is left and ventral is down. Scale bars: (a-d) = 50 μm; (e,f) = 100 μm.

The majority of the 35 selected GS lines affected specific aspects of growth and branching of larval dendrites. The results of our study are catalogued in Table 1. Examples of phenotypes are provided in Figure 2 and are summarized below. Where phenotypic defects were specific to particular classes, we confirmed this with class-specific driver lines (Figure 3).

#### Growth (12 lines)

Dendritic arbors with overtly reduced field area were interpreted as having growth defects. In other GS lines, the field area was unaffected because the major branches extended fully, yet minor branches of higher order showed reduced growth. For example, in class IV ddaC dendrites, misexpression of GSd239 reduced the length, but not necessarily the number of higher order branches (Figure 2d).

#### Branch number (31 lines)

Increases and decreases of branch number were also observed. For example, misexpression of GSd302 (Figure 2b) and GSd454 (Figure 3f,g) specifically reduced the number of higher though not lower order branches.

#### Branch type (six lines)

In class I neurons, GSd458 increased the number of short tips on the normally bare primary and secondary branches (Figures 2f and 3b,g). The opposite effect was caused by misexpression of GSd236, which reduced the number of spine-like protrusions normally found on class III neurons (Figures 2h and 3d,d',g).

#### Degeneration (four lines)

Despite only mild effects on embryonic dendrite morphology, several GS lines caused severely reduced dendritic trees at larval stages, often accompanied by degeneration of the cell soma, for example, GSd402 (Figure 2i,j) and GSd450 (Figure 2k,l).

### Summary of phenotypes induced in da neuron dendrites

In total, we identified 43 GS lines causing da dendrite phenotypes at embryonic (8) or larval (35) stages. The phenotypes generated by many GS lines resemble those of known mutants and fall into five categories that may relate to known molecular pathways (Table 1). In the first category, four GS lines had phenotypes that resemble genetic mutations in *shrub*, which leads to reduced embryonic da dendrite growth [26]. The second category (six lines) resembles phenotypes of *Polycomb Group *genes like *E(z)*, *esc*, or *Su(z)12*, which are involved in the maintenance of dendritic arbors of class IV neurons [27]. The third category contains seven GS lines that showed effects similar to *cut *mutations, the levels of which regulate class-specific dendritic growth and terminal branching [28]. A fourth phenotypic category is represented by GSd484, which resembled *spineless *mutants because it increased class I and reduced class III and IV dendrites [29]. However, *spineless *mutants also have increased class II dendrites, which we did not observe with GSd484. Fifth, GSd496 showed reduced dendritic branching in embryonic da neurons, similar to constitutive activation of the GTPase cdc42 [15].

In addition to these five phenotypic categories, we also identified 24 GS lines causing phenotypes that did not resemble known mutants. These lines suggest the existence of additional genetic pathways that underlie dendritic development and may, via their insertion sites into the genome, provide clues about their molecular nature.

### A gain-of-function screen for genes that affect central neuron dendrites

**Figure 4:**
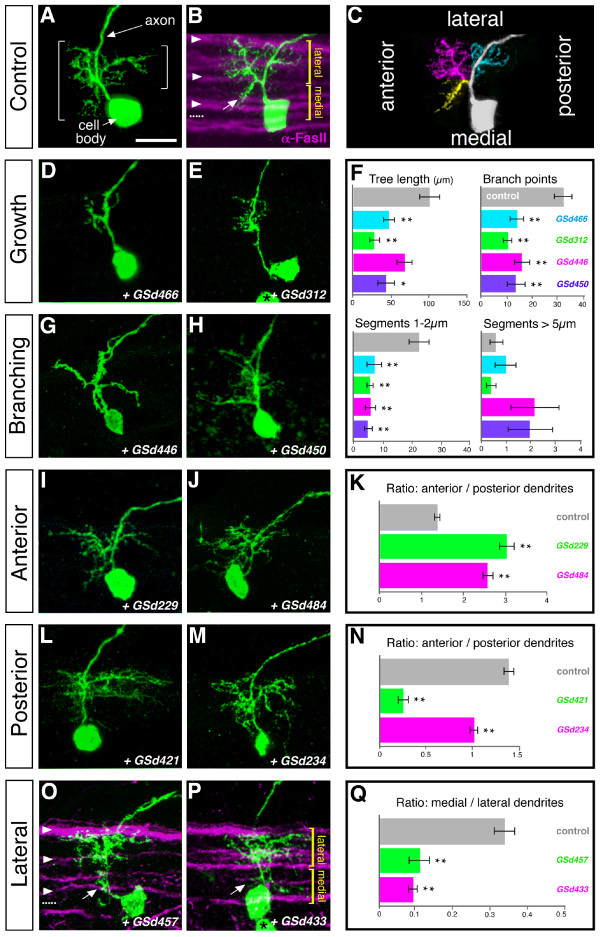
**RP2 dendrite screen – phenotypic categories.** Rows show examples representing the main phenotypic categories recovered from the central (RP2) neuron dendrite misexpression screen. Left and centre columns: confocal images (maximal Z-projections) of RP2 neurons at 25–31 hours AEL, visualised with *UAS-mCD8::GFP*. **(a) **Control RP2 neuron with brackets indicating the dendritic tree. **(b) **Control RP2 neuron in the context of a set of axon tracts visualised by anti-FasciclinII staining (magenta), with arrowheads pointing from top to bottom to the lateral, intermediate and medial FasciclinII tracts and the midline indicated by a dotted line. Dendrites between the lateral and central intermediate Fasciclin II fascicle are defined as 'lateral'; dendrites located between the central intermediate fascicle and the midline as 'medial'; the same applies to (o,p). **(c) **Same neuron as in (b) but with sectors of its dendritic tree pseudo-coloured to highlight branches targeted to anterior lateral (magenta), anterior medial (yellow) and posterior lateral (cyan) regions. Anterior is left and the ventral midline is down. **(d,e,g,h,i,j,l,m,o,p) **Experimental cells: misexpression lines are indicated in the bottom right-hand corner of each panel. Right column: **(f,k,n,q) **quantifications of the dendritic phenotypes shown in the left and central columns. As illustrated in (f), both dendritic tree length and number of branching events are reduced in the 'Growth' and 'Branching' categories. 'Branching' phenotypes have trees with an anterior-posterior extent comparable to controls (Additional file 2) but have an altered pattern of branching: fewer branching events and more segments that are longer (>5 μm). *P < 0.01, **P < 0.005, *t*-test, N = 5. Error bars indicate the standard error. Arrows in (b,o,p) point to medial branches present in controls (b) and absent/reduced in experiments (o,p). Black asterisks in (e,p) indicate the cell body of the contralateral RP2 neuron. Scale bar: 10 μm.

To compare dendrite morphogenesis between peripheral sensory neurons and central neurons, we executed a comparable misexpression screen for central neuron dendrites. Using a new FLPout based system we expressed the same 141 GS lines discussed above in selected RP2 motor neurons, again using mCD8::GFP to reveal dendrite morphologies [22]. With this system, misexpression is initiated by 14–15 hours after egg laying (AEL). At this time, RP2 neurons have begun to establish characteristic dendritic trees in a particular neuropile territory and, in the periphery, RP2 axons have made contacts with their target muscles [30]. We examined RP2 dendritic trees more than 10 hours later, at 25–31 hours AEL, when the majority of dendritic branches are normally located in the lateral neuropile and only a few branches project towards the midline (Figure 4a–c).

We identified 60 GS lines that affect specific aspects of RP2 dendrite development (summarised in Table 2; for a comparative summary of both screens see Additional file 1). The resultant RP2 phenotypes fall into two partially overlapping classes: altered dendritic growth and/or branching; and aberrant dendritic targeting.

**Table 2:** Summary of phenotypes observed in RP2 neurons

			Observed increases (+) or decreases (-)						
				
					Targeting				
						
Line	Cytological location	Closest gene	Growth	Branching	Anterior	Posterior	Medial	Lateral	Expression in CNS: ubiquitous (Ub) or patterned (P)
GSd324	2L;32E2	*ab*			+	-			Not in CNS
GSd332	3R;92F2	*bon*			-	+		+	P
GSd226	2L;21D1	*cbt*			+	-	-	+	Ub
GSd321	3R;91F4	*CG11779*	-		-	-	-	-	P
GSd239	3R;86E11	*CG14709*			-	+	-		P
GSd449	3R;86E11	*CG14709*			+	-	+		P
GSd440	2R;43D1	*CG1602*				-			Ub
GSd450	2L;38D5	*CG2617*		-					P
GSd454	2L;30B5	*CG33298*	-			-	-	-	Ub
GSd466	2L;30B5	*CG33298*	-		-	-	-	-	Ub
GSd486	2R;58D4	*CG3624*			Variable	Variable			P
GSd499	3L;74E2	*CG7510*	-		-	-	-	-	P
GSd211	3R;87D7	*CG7518*	-		-	-	-	-	P
GSd098	2R;49B12	*CG8776*			-	+	-	+	Ub
GSd496	2L;29F8	*CG9582*		-					Ub
GSd322	3R;90D1	*cpo*			+	-			P
GSd447	2L;36C9	*dl*	-		-	-	-	-	P
GSd066	3L;61B3	*E(bx)*			-	+	-	+	P
GSd451	3L;61B3	*E(bx)*			-	+			P
GSd492	3L;61B3	*E(bx)*			+	-	-		P
GSd017	3L;75B2	*Eip75B*		-	+	-			Ub
GSd436	2L;35D2	*esg*			-	+	-	+	P
GSd481^a^	2L;35D2 2R;55C8	*esg imd/Dp1*	-		-	-	-	-	P, imd N/D, Dp in CNS (Ub)
GSd421	3R;100C2	EST:LP08211			-	+	-	+	N/D
GSd207	2R;53D11	EST:SD02913		-	-	+			N/D
GSd233	3R;88A4	*foxo*				+	-	+	Ub
GSd406^a^	3R;88A5 2L;26C4	*foxo slam (antisense)*				+	-	+	Ub
GSd445	2L;36A10	*grp*			+	-	-		Ub
GSd410	3L;66C13	*Gug*			-	+	-	+	Ub [96]
GSd312	3R;99E4	*hdc*	-		-	-	-	-	P
GSd404	3R;99E4	*hdc*	-		-	-	-	-	P
GSd457	2R;57F10	*HmgD*				+	-	+	P
GSd327	2L;38E3	*Hr38*			+	-	-		Ub
GSd031	2R;55C4	*IM1*			+	-			Ub
GSd056	3L;80A4	*jim*			+	-			Ub
GSd482	2R;60A6	*ken*			Variable	Variable			P
GSd204	2L;26B5	*Kr-h1*				+			P [97]
GSd433	2L;22A1	*lea (Robo2)*			-	+	-	+	P
GSd057	2R;44A4	*lig*			+	-	-		P
GSd424	3L;76B9	*lush*			-	+			N/D
GSd427	2R;50C23	*mam*	-	-	-	-	-	-	P [97]
GSd456	3R;96A9	*mld*			-	+			P
GSd484	3R;93D9	*mod(mdg4)*			+	-	+		P
GSd314	3R;96E2	*msi*			+	-	-		P
GSd302	3R;91F4	*nos*	-		-	-	-	-	Not in CNS
GSd201	3R;94E13	*pnt*			+	-			P [47]
GSd229	3R;94E10	*pnt*			+	-	+		P [47]
GSd458	2R;47A13	*psq*					-		P
GSd231	2R;52E5	*Rho1*				+	+		Ub
GSd472	2R;47D6	*shn*		-	-	+			Ub
GSd248	2L;21B3	*spen*			+	-	-	+	Ub
GSd500	3L;70D7	*stwl*			-	+			Ub
GSd234	2R;49E7	*Su(z)2*				+			P
GSd426	2R;49E7	*Su(z)2*			-	+	-	+	P
GSd309	3R;89B9	*tara*					+	-	Ub
GSd413	3R;89B8	*tara*			-	+			Ub
GSd485	3R;89B9	*tara*			+	-	-		Ub
GSd446	3R;100D1	*ttk*		-					Not in CNS neurons [98]
GSd468	3R;100D1	*ttk*		-					Not in CNS neurons [98]
GSd462^a^	3R;100D1 3R;92F1	*ttk Stat92E*		-					Not in CNS neurons [98]

^a^Lines GSd481, GSd406, and GSd462 each harbour two insertions of the GS element.

#### Growth and branching (19 lines)

**Figure 5:**
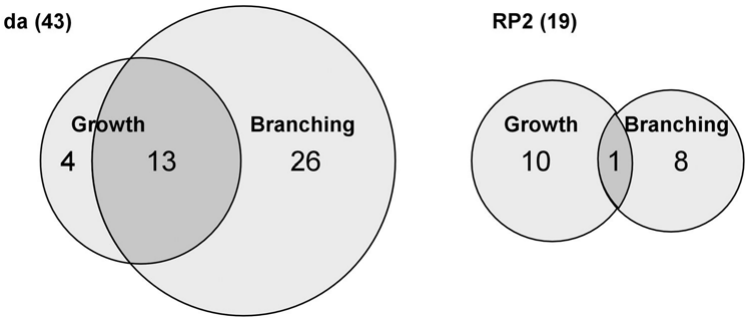
**Dendrite growth and branching are distinctly affected by gene misexpression.** Proportional Venn diagrams to show degree of overlap among lines with effects on dendrite growth and/or branching.

Ten lines affected dendritic growth, reducing field size. We quantified the phenotypes caused by two lines (GSd466 and GSd312) in detail and found that the reduction in overall dendritic tree length was linked to a reduction in the number of branch points (Figure 4d–f). Eight lines caused branching defects, altering the pattern of branching. While this appears to be associated with reduced growth and branch point number (Figure 4f), the category of 'branching' phenotypes can be distinguished from 'growth' phenotypes based on the extent to which the dendritic trees span neuropile territories in the antero-posterior axis (Additional file 2). Quantification of the phenotypes caused by expression of GSd446 and GSd450 further showed that the altered pattern of branching produced more long (>5 μm) dendritic segments, as is typical for other motor neuron classes (Figure 4f–h; M Tripodi *et al*., submitted). Only one line (GSd427) affected both growth and branching. It is possible that in some additional cases reduced growth could have disguised an effect on branching. Unlike peripheral (da) dendrites, we did not find any lines that caused overgrowth of RP2 dendrites. Similar to da dendrites, the regulation of RP2 dendritic growth and branching are clearly genetically separable (Figure 5).

#### Targeting in the antero-posterior axis (40 lines)

17 GS lines led to an extended anterior dendritic field and a trimming of the posterior field (Figure 4i–k). 22 lines induced an enlarged posterior territory, frequently (16/22) with reductions in the anterior dendritic field (Figure 4l,m and Table 2). One line, GSd440, led to reductions of the posterior tree only (not shown).

#### Targeting in the medio-lateral axis (26 lines)

To evaluate the medio-lateral distribution of dendrites, we used the set of FasciclinII axon bundles as landmarks [31] and defined the neuropile between the lateral and central intermediate axon bundle as 'lateral', and the region between the central intermediate fascicle and the ventral midline as 'medial' (Figure 4b,o,p). 20 GS lines caused reductions of medial branches, often (12/20) also increasing lateral branches (Figure 4f–q). Five lines induced extra medial branches, though only one of these, GSd309, showed concomitant reductions of lateral dendrites (Table 2).

In the majority of cases (70%), we found that dendritic mis-targeting led to a shift of the dendritic territory within a neuropile axis, as expansion in one direction was accompanied by a complementary reduction in the other. Expansions of the dendritic field in one direction only were much less frequent (30%).

### Dendritic targeting along Cartesian co-ordinates

Of the various aspects of dendritic development, directed growth into a particular territory is arguably least understood and few genes required for this process have been identified [6,32-36]. Dendritic targeting phenotypes recovered in this screen can be sorted into four categories: shifts of the dendritic territory to the anterior, posterior, medial or lateral. These categories are compatible with a model of dendrites being targeted along Cartesian co-ordinates. The existence of distinct dendritic domains in the antero-posterior neuropile axis has been illustrated previously [30]. The phenotypes of this screen further suggest that motor neuron dendrites might also be patterned with respect to the ventral midline, along the medio-lateral neuropile axis.

**Figure 6:**
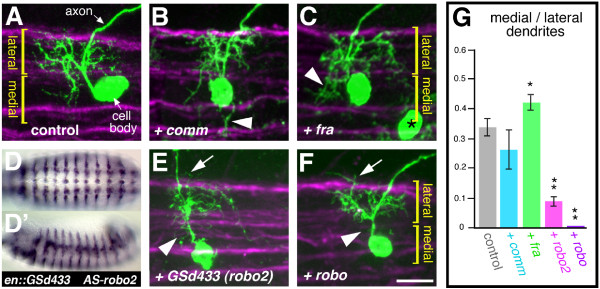
**Dendritic targeting relative to the ventral midline.(a) **Control and **(b,c,e,f) **experiments showing confocal images (maximal Z-projections) of RP2 neurons at 25–31 hours AEL, visualised with *UAS-mCD8::GFP *(green) in the context of a set of axon tracts visualised by anti-FasciclinII staining (magenta). Dendrites between the lateral and central intermediate Fasciclin II fascicle are defined as 'lateral'; dendrites located between the central intermediate fascicle and the midline as 'medial'. Misexpression lines are indicated in the bottom left hand corner of each panel. **(b) **Misexpression of *commissureless *(*comm*) leads to aberrant midline crossing of dendritic branches (arrowhead), though no apparent increase of dendrites targeted towards the midline between the intermediate and medial FascilinII tracts. The high variability in phenotype is partly due to the varying lengths the dendritic tree mis-routed across the ventral midline. **(c) **Misexpression of *frazzled *(*fra*) causes increased targeting of dendrites into the medial neuropile (arrowhead). Black asterisk indicates the cell body of the contralateral RP2 neuron. **(d,d') **Ventral (d) and lateral (d') views of stage 13 embryos driving expression of GSd433 with *engrailed-GAL4 *and stained by in *situ *hybridisation using an anti-sense probe against *robo2*. The staining shows the segmentally repeated stripes characteristic for *engrailed*. The reaction had to be terminated before the endogenous *robo2 *expression pattern appeared (see Additional file 3) due the high levels of expression. **(e,f) **Misexpression of *robo2 *by GSd433 (e) or *robo *(f) leads to a reduction to near absence (*robo*) of branches innervating the medial neuropile (arrowheads), and some dendritic branches positioned aberrantly lateral of the lateral Fasciclin II axon tract (arrows). **(g) **Quantification showing ratios of medial/lateral dendrites; *P = 0.04, **P < 0.001, *t*-test, N = 5; error bars indicate the standard error. Anterior is left. Scale bars: (a-c,e,f) = 10 μm; (d,d') = 140 μm.

Targeting relative to the ventral midline has been documented for axons [37,38]. Some of the molecular cues involved in this process (Slit and Netrins) and their receptors (Robo, Robo2, Robo3 and Frazzled/DCC) have also been shown to regulate midline crossing of dendrites in the *Drosophila *nerve cord [33,34]. We found that a GS insertion near *lea/robo2*, which encodes a receptor for the midline repulsive cue Slit [37,38], caused a reduction of dendrites innervating the medial neuropile (Figure 6e). We therefore asked if midline-derived guidance cues such as Slit and Netrin might be involved in targeting dendritic trees to distinct medio-lateral territories, in addition to their documented role in midline crossing. To test this hypothesis we over-expressed other elements of the Slit and Netrin signalling pathways: *robo*, *commissureless *and *frazzled*. As previously reported, expression in RP2 of the *robo *antagonist *commissureless *[39,40] or *frazzled *could lead to inappropriate midline crossing of RP2 dendrites [33]. However, we found that expression of *frazzled*, though not *commissureless *(N = 49), also led to an expansion of the RP2 dendrites innervating the medial neuropile (Figure 6a–c,g), which is normally occupied by dendrites of other motor neurons such as RP1 and RP3. Conversely, expression of the Slit receptor Robo led to an absence of medially positioned branches (Figure 6f,g).

Our results support the idea that dendrites in the CNS are targeted along the antero-posterior and medio-lateral neuropile axes using, at least in part, guidance cues that also pattern axon trajectories.

### Growth and branching of central dendrites is specified independently from the target territory

**Figure 7:**
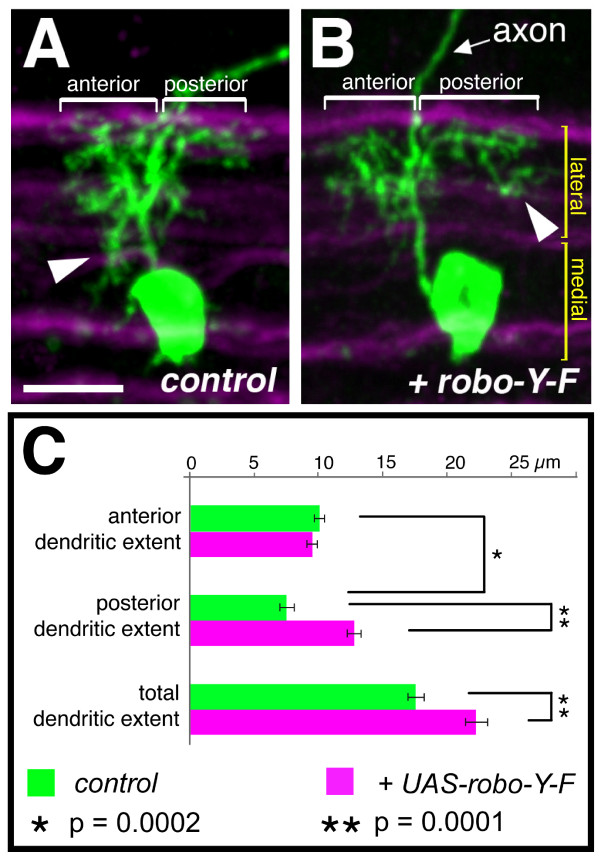
**Displacement of dendritic branches from medial anterior to posterior lateral regions. **RP2 neurons at 25–31 hours AEL and visualised with *UAS-mCD8::GFP *in the context of FascicilinII positive axon bundles (magenta) demarcating the medial and lateral neuropile (maximal Z-projections of confocal image stacks). **(a) **Control. **(b) **Misexpression of *UAS-robo-Y-F *(activated *robo*) leads to a lack of dendritic innervation of the medial neuropile (normally located anterior to the axon (arrowhead in (a)) and a concomitant expansion of dendrites in the lateral neuropile posterior to the axon (arrowhead in (b))). Dendritic extent anterior or posterior to the axon is indicated by brackets. **(c) **Quantification of anterior, posterior and total (combined) maximal dendritic extent for controls (green, N = 10) and *UAS-robo-Y-F *expression RP2 neurons (magenta, N = 8). The significance of pair-wise comparisons using Student's *t*-test is indicated. Anterior is left and the ventral midline is down. Scale bar: 20 μm.

We next investigated the strategy with which motor neuron dendrites innervate particular neuropile territories. Do dendritic arbors expand until their target territory is occupied? Or do motor neurons have a program of dendritic growth and branching that is independent of the positioning of dendrites within the neuropile? To distinguish between these alternatives, we altered the dendritic territory of RP2 by misexpression of an activated form of Robo (Robo-Y-F [41]). This manipulation suppressed the establishment of dendritic branches in the medial neuropile anterior of the axon and led to a concomitant posterior expansion of the arbor, phenocopying GS lines that also reduce the medial dendritic territory (Figure 7). We measured the maximal distances (extent) to which dendritic trees extend anterior and posterior of the axon. We found that this manipulation led to a significant increase in the extent to which RP2 dendrites project posteriorly (dendritic extent of posterior arbors: 7.4 ± 1.5 μm wild type versus 12.7 ± 1.3 μm Robo-Y-F, *p *= 0.0001, *t*-test; Figure 7). However, expression of *UAS-robo-YF *does not abolish the establishment of anterior dendrites in the lateral neuropile and the anterior extent of the arbor is therefore comparable to controls (anterior arbors: 10 ± 0.9 μm wild type versus 9.4 ± 1 μm Robo-Y-F, *p *= 0.22, *t*-test; N = 8; Figure 7). This correlation between the induced absence of branches in the medial anterior neuropile and the extension of the posterior territory is compatible with the notion that expression of *UAS-robo-YF *may have caused a displacement of part of the dendritic tree from a medial anterior to a lateral posterior domain.

**Figure 8:**
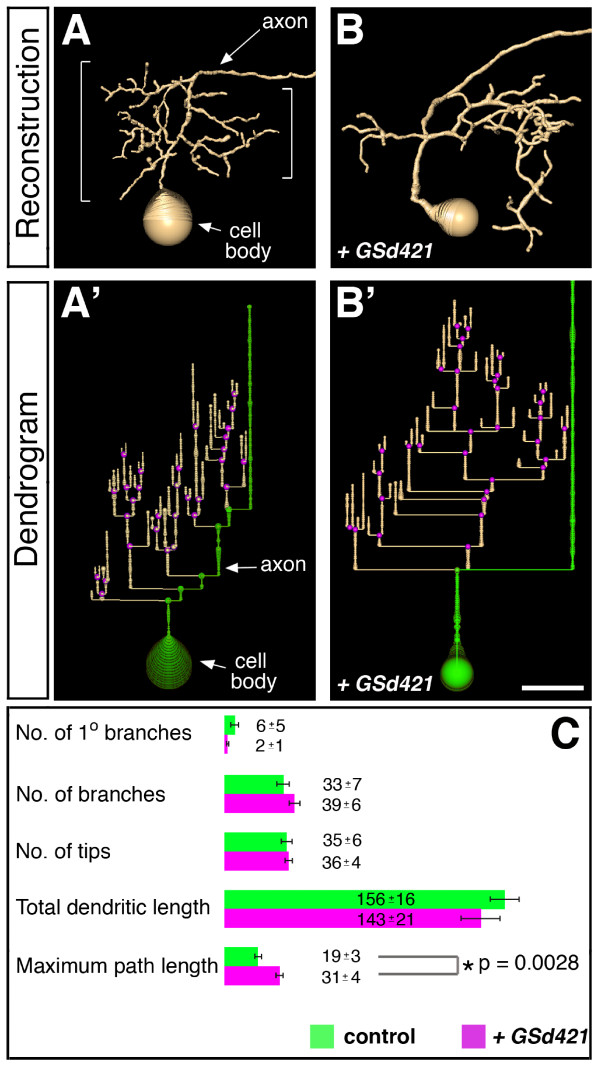
**Dendritic growth, branching and targeting are regulated independently. (a,b) **Three-dimensional reconstructions from confocal image stacks of RP2 neurons at 25–31 hours AEL and visualised with *UAS-mCD8::GFP *generated with AMIRA software. **(a) **Control. **(b) **Misexpression of *GSd421 *causes aberrant dendritic targeting to the posterior. Brackets in (a) indicate the dendritic tree. **(a',b') **Dendrograms derived from the reconstructions with branch points highlighted in magenta and the cell body and axon offset from the dendritic tree by green. **(c) **Quantification of the dendritic architectures for controls (green, N = 4) and *GSd421 *expressing RP2 neurons (magenta, N = 4). The significance of pair-wise comparisons using Student's *t*-test is indicated. Error bars indicate the standard error. Anterior is left and the ventral midline is down. Scale bar: 10 μm.

To further investigate the relationship between dendritic growth and targeting, we compared controls to RP2 neurons with marked dendritic mistargeting phenotypes as induced by misexpression of GSd421 (Figure 8). For a quantitative readout we reconstructed dendritic trees from three-dimensional confocal image stacks using recently developed reconstruction algorithms [42,43] (Figure 8a,b). As anticipated, we found significant differences in the directionality of dendritic growth (that is, targeting), reflected by the maximum dendritic path lengths from the axon to the perimeter of the dendritic trees (19 ± 3 μm control versus 31 ± 4 μm experimental, *p *< 0.003, *t*-test) (Figure 8c). However, control and GSd421-misexpressing RP2 neurons did not differ significantly in other aspects of dendritic growth and branching, including total dendritic length (157 ± 16 μm wild type versus 143 ± 21 μm GSd421) and number of dendritic tips generated (35 ± 6 wild type versus 36 ± 4 GSd421).

These observations demonstrate that dendritic growth and branching are regulated by mechanisms that are genetically separable from targeting: RP2 motor neurons generate a set quantity of dendritic length and branches independent of the neuropile domains in which they are positioned. The quantitative analysis further addresses the longstanding issue of which attributes of neurons are genetically specified and which are subject to non-genetic influences and, therefore, highly variable [44,45]. Here we show that total dendritic length, branch point number and territories of branching are reproducible features. In contrast, the number of primary branches was highly variable.

### Reliability of the misexpression screens

Next, we assessed the reliability of the screening method by making use of the fact that, for each of the GS lines, the closest gene predicted to be expressed in response to Gal4 has previously been identified [23]. First, in the entire collection of 141 lines, there were 29 genes for which there were at least two independent GS insertions. We determined the frequency with which independent GS-lines near the same gene gave concordant results: 20/29 genes (69%) in the da screen and 17/29 (59%) in the RP2 screen (Tables 1 and 2). Where different phenotypes were induced by the expression of independent GS lines near the same gene, this may in some instances be due to insertion site-specific variations in gene expression levels. In other cases, different GS insertions in the same gene may generate functionally distinct transcripts, as is predicted for insertions in the *Kr-h1 *(GSd113 = *Kr-h1-RA*; GSd204 = *Kr-h1-RB *transcript) and *pnt *loci (GSd229 = *pnt-RC*; GSd236 and GSd420 = *pnt-RB *transcript) [46-48]. With 69% and 59% concordance, respectively, and evidence for isoform-specific insertions at some of the discordant loci, we judge these screens to be a reliable means for identifying genes that influence dendrite development.

Secondly, we asked if the screens reported here had recovered predicted candidate genes. Among the 35 genes identified in the da screen, five have been studied previously in the context of da dendrite development: *abrupt *(*ab*), *nanos *(*nos*), *bonus *(*bon*), *E(bx) *and *tramtrack *(*ttk*). In all of these cases, the GS misexpression phenotypes are conversely related to the reported mutant or RNA interference (RNAi) knockdown phenotypes [11,49-51]. For instance, mutation or RNAi knockdown of *ab *increases arborisation of class I neurons, while expression of GSd324 (inserted closest to *ab*) in class II-IV da neurons reduces dendritic branching as previously reported for misexpression of *ab *[50,51]. This concurrence suggests that the additional 30 candidate genes identified in the da screen may reveal new molecular determinants of PNS dendrite morphologies.

For the screen on central neuron dendrites we had to gauge its utility differently, since genes regulating the development of RP2 dendrites remain largely unknown. We therefore examined with *in situ *hybridization whether identified genes were actually expressed in the embryonic CNS during the time of dendritic outgrowth, stages 14–16. We assayed 47 of the 51 genes and found 43 to be expressed in the CNS, 25 of these in subsets of cells (Table 2; Additional file 3). These expression data suggest a high degree of confidence in the validity of the screen, though further loss-of-function studies are needed to test this directly.

### A comparison of dendrite development between peripheral and central neurons

**Figure 9:**
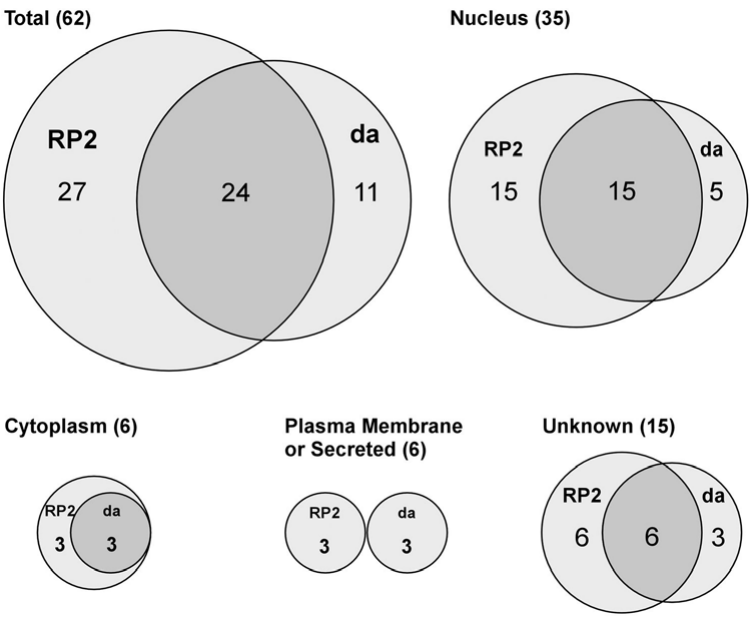
**Overlap of RP2 and da screens, classified by sites of gene activity. **Proportional Venn diagrams to describe the degree of overlap among genes that emerged from both screens. The total is shown at top left, and then broken down by the predicted site of gene product activity.

One of our aims was to ask whether the development of peripheral and central neuron dendrites is influenced by common or distinct molecular mechanisms. We did so by comparing the effects of the same GS lines on da and RP2 neurons. Of the 35 unique genes identified in the da screen and 51 for RP2, there were 24 genes (39%) that were capable of influencing dendrites in both cell types (Table 3; Figure 9). Therefore, there were 11/35 genes (31%) that were particular to the da screen, while 27/51 genes (53%) were specific to the RP2 screen. This provides evidence that neither screen was prone to chronically low levels of gene misexpression that might prevent detection of phenotypes, and that each screen could reveal unique genes.

**Table 3:** Genes closest to the GS insertions that cause dendrite phenotypes

	Phenotype		
		
Proposed site of protein activity	RP2	da	Molecular function
**Nucleus**			
*ab*	•	•	BTB/POZ domain transcription factor
*apt*		•	bZIP transcription factor, RNA binding
*bl*		•	KH domain protein, RNA binding,
*bon*	•	•	Nuclear receptor cofactor
*cbt*	•	•	C2H2 zinc finger transcription factor
*cpo*	•		RRM-motif protein
*dl*	•		NFkappaB-like transcription factor
*Dp1*	•		Multi-KH-domain DNA binding protein
*E(bx)*	•	•	ISWI-containing chromatin remodelling protein
*Eip75B*	•		Nuclear hormone receptor
*esg*	•		Zinc-finger transcriptional repressor
*foxo*	•	•	Forkhead transcription factor
*grp*	•		Serine.threonine checkpoint kinase
*Gug*	•		Atrophin-like transcription regulator
*HmgD*	•		Chromatin remodeling protein
*Hr38*	•	•	Nuclear hormone receptor
*IM1*	•		Immune-induced molecule
*jim*	•		Zinc-finger protein
*ken*	•	•	BTB/POZ domain transcription factor
*Kr-h1*	•	•	Zinc-finger protein
*lola*		•	BTB/POZ domain transcription factor
*mam*	•		Transcriptional coactivator
*mir-313*		•	microRNA
*mld*	•		Zinc-finger protein
*mod(mdg4)*	•	•	BTB/POZ domain transcription factor
*msi*	•		RNA binding protein
*pnt*	•	•	ETS domain transcription factor
*psq*	•	•	BTB/POZ domain transcription factor
*shn*	•	•	Zinc-finger protein
*Stat92E*	•	•	Transcription factor
*stwl*	•	•	Transcription factor
*Su(z)2*	•		Zinc finger protein
*tara*	•		Nuclear protein of trithorax group
*ttk*	•	•	BTB/POZ domain transcription factor
*woc*		•	Zinc-finger transcription factor
			
**Cytoplasm**			
*hdc*	•	•	Cysteine-rich cytoplasmic protein
*imd*	•		Death domain adaptor protein
*lig*	•		Novel protein
*nos*	•	•	Translation factor
*Rho1*	•	•	GTPase
*spen*	•		RRM-motif protein
			
**Plasma membrane**			
*for*		•	Cyclic nucleotide-dependent kinase
*slam *(*antisense*)	•		Novel protein
*lea *(*Robo2*)	•		Transmembrane receptor
*PFE*		•	Transmembrane receptor kinase
			
**Secreted**			
*bnl*		•	Growth factor
*lush*	•		Odorant binding protein
			
**Unknown**			
*CG11779*	•	•	
*CG11897*		•	
*CG14709*	•	•	
*CG1602*	•		
*CG2617*	•	•	
*CG33298*	•	•	
*CG33558*		•	
*CG3624*	•		
*CG7510*	•		
*CG7518*	•	•	
*CG8776*	•		
*CG9582*	•	•	
EST:EN05557/EN06658		•	
EST:LP08211	•		
EST:SD02913	•		

We classified the products of all previously studied genes according to their proposed site of activity (nucleus, cytoplasm, plasma membrane, secreted; Figure 9). The majority (56%) are predicted to encode nuclear proteins. This is not surprising since the lines in the GS collection were pre-selected for lethal effects when expressed throughout the nervous system. We have argued previously that this pre-selection, which has enriched the collection for visible phenotypes, may also have biased the collection toward transcriptional regulators whose misexpression may deregulate the expression of multiple downstream genes [23]. Such factors could also interfere with the establishment of cell fate in some cases, causing transformations that switch dendritic architecture toward that of other classes. The proportion of genes encoding nuclear factors that were either unique or common to the two screens is similar to the proportions observed for all sites of activity in total (Figure 9). The same is true for unknown proteins (Figure 9). Notably, all of the cytoplasmic proteins that we identified to have an effect in the da screen also affected RP2 dendrites. This analysis suggests that peripheral and central dendrites are influenced by partially overlapping cytoplasmic and nuclear regulatory programs. However, the complete lack of overlap among the plasma membrane-associated and secreted proteins (Figure 9) suggests that factors mediating interactions between developing dendrites and substrata may be highly specific for peripheral versus central neurons.

### The EcR regulates the morphogenesis of da neuron dendrites

Finally, we wanted to test whether there were molecular mechanisms uncovered by these screens that were required for dendrite morphogenesis, and whether these mechanisms shed light on common or distinct pathways for peripheral and central dendrites. In both the da and RP2 screens, we identified several candidate genes (*Kr-h1*, *bon*, *Hr38*) related to signalling from nuclear hormones and particularly ecdysone (Additional file 4). In insects, ecdysone initiates major developmental transitions and regulates dendrite regression, pruning and re-growth among sensory da neurons and motor neurons, as well as central mushroom body neurons and peptidergic neurosecretory neurons [9,52-57]. Kr-h1 is a stage-specific modulator of the prepupal ecdysone response [46], and ectopic Kr-h1 dramatically reduces dendrite branching in da neurons (this study, GSd113) and mushroom body neurons [58]. *bon *codes for a transcription factor regulating genes involved in ecdysone responses [59], and *Hr38 *encodes an orphan receptor that can compete with the ecdysone receptor (EcR) for binding to its obligate co-receptor, Ultraspiracle (Usp) [60,61].

Our findings that *Kr-h1*, *bon *and *Hr38 *may influence the morphologies of da dendrites suggested a new role for ecdysone signalling in addition to its role in metamorphosis when it induces dendrite regression and pruning. This is consistent with a recent study that showed that arborisations of class I da neurons are reduced by RNAi knockdown and mutations in *EcR *and *usp *[11]. However, it remained unresolved whether EcR and Usp are required cell-autonomously in da neurons, as is the case for dendrite pruning at pupariation [9,54].

**Figure 10:**
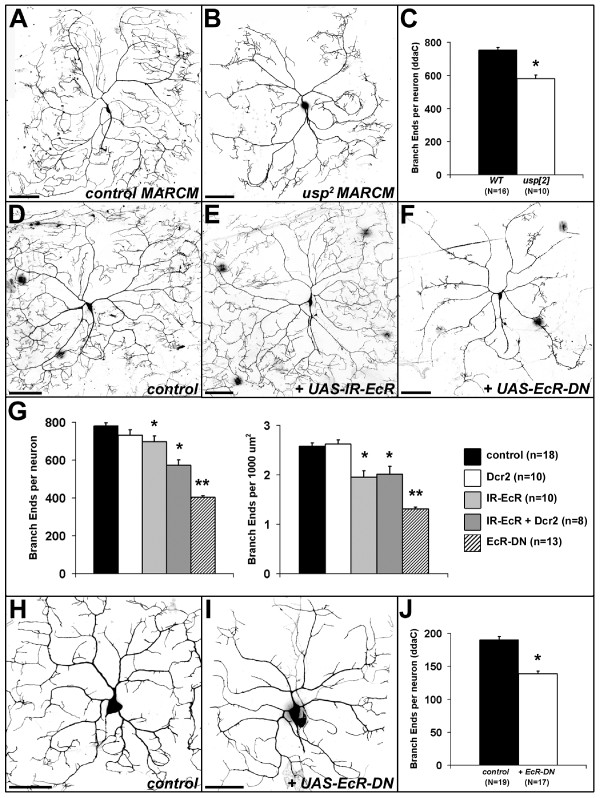
**The EcR pathway is required for peripheral dendrite development.(a) **Control class IV ddaC MARCM clone. **(b) ***usp*^2 ^MARCM clone showing reduced ddaC dendrite branches. **(c) **Quantification of the mean number of branch ends per neuron, comparing wild-type (WT) to *usp*^2 ^MARCM clones. The asterisk indicates significant reduction (*t*-test, *P *< 0.000001). **(d) **Control ddaC neuron (genotype: *UAS-mCD8::GFP/+;;ppk1.9-GAL4/+*). **(e) **Expression of RNAi-inducing *UAS-IR-EcR*, targeting all EcR isoforms. **(f) **Expression of a dominant-negative EcR (EcR-DN). **(g) **The graph on left shows the mean number of branch ends per neuron for all genotypes tested, including those co-expressing *UAS-Dicer2 *(Dcr2), a component of the RNAi machinery that can potentiate the RNAi effect [88]. The graph on right shows the mean branch density in ddaC class IV neurons. Pairwise comparisons (ANOVA, Tukey, *P *< 0.0001) determined that EcR RNAi significantly reduced both branch number per neuron and branch density (single asterisks). EcR-DN further reduced branch number and density to levels lower than both controls and RNAi (double asterisks). The analysis revealed that the RNAi-induced reduction of branch density (right graph) was not enhanced by coexpression of Dcr2. **(h) **Control ddaC neuron (same genotype as (d)) in first instar larva (28–30 hours AEL). **(i) **Expression of EcR-DN (same genotype as (f)). **(j) **EcR-DN reduced branch number in first instar larvae (asterisk, *t*-test, *P *< 1e^-7^), but did not influence the field area (control = 11,349.7 ± 324.6 μm^2 ^versus EcR-DN = 12,261.0 ± 372.7 μm^2^, *t*-test, *P *= 0.07). Error bars in (c,g,j) indicate standard error. Anterior is left and ventral is down. Scale bars: (a,b,d-f) = 100 μm; (h,i) = 25 μm.

To investigate this further, we confirmed expression of the EcR-A and EcR-B1 protein isoforms [62] in dorsal da neurons of third instar larvae (Additional file 5) [54]. Focusing on the class IV neuron ddaC, we then tested with three approaches whether there is a requirement for the EcR in ddaC dendrite development. First, since mosaic analysis with a repressible cell marker (MARCM) could not be applied directly to the EcR due to its cytological location, we generated MARCM clones for mutations in the EcR co-receptor, Usp [9,63]. Relative to control clones (Figure 10a), the dendritic arbors of ddaC neurons in *usp*^2 ^null mutant clones had reduced numbers of branches (control = 754.1 ± 15.1 versus *usp*^2 ^MARCM = 580.3 ± 23.1; *p *< 1e^-6^, *t*-test; Figure 10b,c). We also examined MARCM clones for *usp*^3 ^(a hypomorphic allele) and *usp*^5^, a missense mutation in the second zinc finger of the DNA binding domain of Usp [9]. Unlike the null *usp*^2 ^allele, neither *usp*^3 ^nor *usp*^5 ^showed branching defects in ddaC neurons (not shown), likely due to residual Usp function in these mutant clones. Second, we used the class IV-specific driver *ppk1.9-GAL4 *[17] to express an RNAi-inducing construct of the EcR (*UAS-IR-EcR*) that targets all EcR isoforms [64]. This resulted in a significant reduction in the mean density of branches to 75.8% of control levels, as measured by the number of branch ends normalised to dendritic field area (Figure 10e,g). Third, as an additional direct manipulation, we expressed a dominant negative form of the receptor (EcR-DN) that binds Usp normally, but fails to bind ecdysone and cannot activate transcription, and is thereby a competitive inhibitor of all endogenous isoforms of EcR [65]. This also reduced the density of ddaC dendrites (50.8% of controls; Figure 10f,g), affecting primarily higher order branches and so having negligible impact on the field area (mean area in controls = 304,899 ± 7,115 μm^2 ^versus EcR-DN = 308,802 ± 7,400 μm^2^).

Together, the reduced branching observed in *usp*^2 ^MARCM clones and in the direct EcR manipulations using RNAi and EcR-DN support the idea that ecdysone promotes the arborisation of developing ddaC neurons prior to and in addition to its role in metamorphosis. This is consistent with other experiments showing that ecdysone enhances neurite outgrowth of cultured *Drosophila *neurons *in vitro *[66]. We explored whether this role for the EcR could influence dendrite arborisation in embryonic and early larval development, or whether it was restricted to later larval life. We found that the EcR was expressed in embryonic dorsal da neurons, including ddaC (Additional file 5), and that reduced branching caused by EcR-DN was already apparent in first-instar larvae 28–30 hours AEL (mean branches per ddaC neuron in controls = 190.4 ± 5.0 versus EcR-DN = 139.1 ± 4.1; Figure 10h–j).

Central RP2 dendrites were also affected by GS lines near the three genes *Kr-h1*, *bon*, and *Hr38 *(Additional file 4). However, unlike in da neurons, misexpression of EcR-DN in the RP2 neuron using the FLPout system did not consistently affect its dendrites despite high expression levels (not shown), suggesting that EcR may not play a role in CNS dendrite growth in embryonic and early larval stages.

## Discussion

The development of dendrites, including their patterns of growth, branching and targeting, are critical to the function of neurons and neural circuits [1]. Here, we have applied genetic screens in *Drosophila *to improve our understanding of cellular and molecular mechanisms governing dendrite development. Specifically, we used a well-characterised collection of 141 GS misexpression lines to perform two gain-of-function screens, one for peripheral neurons and one for central neurons. Our goals were: to identify new candidate genes involved in dendrite development; to understand better the organizational framework within which dendrites develop; and to compare dendrite development between peripheral and central neurons through the candidate genes identified and their misexpression phenotypes.

### Identification of genes involved in dendrite patterning

This study is the first published report of a misexpression approach used to identify candidate genes required for dendrite development. Clearly, this strategy has limitations since genes that induce phenotypes may not be required for dendrite development, nor are the phenotypes necessarily informative. Despite these concerns, we judge these gain-of-function screens a useful alternative to forward loss-of-function genetic screens, which can be limited by technical challenges (for example, when targeting specific cells) and by genetic redundancies. We deem it a valid strategy for gene discovery in many cases since at least 5 of the 35 genes identified in the da neuron screen (*ab*, *nos*, *bon*, *E(bx) *and *ttk*) are known to be required for da dendrite patterning and have loss-of-function phenotypes that are the converse of the gain-of-function phenotypes described here [11,49-51]. Notably, the da screen led to the discovery that signaling through the EcR pathway promotes the arborisation of developing da neurons prior to metamorphosis.

Considerably more candidate genes, 51, were identified in the central neuron (RP2) dendrite screen. This difference in sensitivity between the two screens may reflect true differences in developmental robustness, or a bias within the GS collection, or technical issues such as the strength of the Gal4 drivers or the ease of detection of mutant phenotypes. The collection did not contain any genes shown previously to be required for dendrite development in central neurons (for example, *shot/kak*,*fra*,*robo*, *sema1a*, *Dscam*) [32-34,36,67]. We were therefore unable to validate the RP2 screen in the same manner as the da neuron screen. However, the phenotypes recovered were specific for particular aspects of dendrite growth, branching and targeting, and as such reveal the constraints of the underlying organizational framework. By way of verification we were able to show that at least 43 of 51 candidate genes from the RP2 screen are expressed in the CNS during the period of dendritic outgrowth and targeting. Since their ectopic and/or elevated expression in RP2 is sufficient to mis-pattern RP2 dendrites, these genes could encode novel factors required for CNS dendrite development.

### Features of dendrite morphogenesis that are genetically regulated: a comparison of peripheral and central dendrites

The second aim of this study was to gain a better understanding of the organizational framework that underlies dendrite development. In both screens the misexpression phenotypes fell into specific categories, demonstrating that specific features of dendrite development, such as growth, branching and targeting can be reproducibly and selectively modulated. Within each screen, there was incomplete overlap between phenotypic categories, suggesting that these features are derived from molecularly distinct mechanisms. Comparing the two screens, we found molecular and phenotypic evidence for similarities as well as differences between peripheral da and central RP2 neurons in the implementation of dendrite growth, branching and targeting.

#### Growth and branching

Both screens produced a segregation of dendritic growth and branching phenotypes, suggesting these processes to be genetically separable for peripheral (da) and central (RP2) neurons (Figure 5). For da neuron dendrites, our data are in agreement with previous studies that identified genes regulating either growth or branching [11,15,26,28,49]. For example, mutations in *shrub *reduce dendritic growth in da neurons [15,26], while mutations of *abrupt*, *cut*,*spineless *or *knot/collier *regulate patterns of branching [28,29,50,51,68-70]. In our screen, we found additional clear examples such as GSd422, which caused increased formation of higher order branches while leaving the growth and pattern of lower order dendrites intact.

How is the extent of growth and branching regulated? For peripheral da neurons we found that many GS lines led to a reduction of either dendrite growth (17 of 43) and/or branching (32 of 43). We also recovered phenotypes with increased growth and branching (for example, GSd422 (Figure 1f) and GSd458 (Figure 2f)). Increased growth and branching has also been reported for *flamingo *and *sequoia *mutants (growth) or mutations in *abrupt *and over-expression of *cut *(branching) [28,50,51,71,72]. Together these observations indicate that for da neurons the rates of growth and branching are not maximal during normal development but are tightly regulated. This regulation is clearly influenced by class-specific factors, as shown here and elsewhere [28,50,51], and by global cues such as ecdysone, which may implement matching the density of da dendrites to the area of growing receptive fields.

In addition, there are also genetic components that link dendritic growth and branching complexity. For instance, we found that two GS lines (GSd231, GSd431) closest to *Rho1 *cause exuberant dendritic branching but at the same time reduce growth. Such opposing effects on growth and branching have also been reported for several transcription factors [11]. The factors that balance growth and branching may be part of a molecular switch that modulates cytoskeletal dynamics to favour growth at the expense of branching or vice versa. Switching between extension and branching may aid the exploratory dendritic growth necessary for dendritic tiling and self-avoidance [25,73-77].

How is dendritic growth and branching regulated in central (RP2) neurons? The quantitative comparison between control and GSd421-expressing RP2 neurons revealed that parameters such as total dendritic length and branch point number are probably specified by the RP2 genetic program of differentiation, since these are fairly invariant features. The detailed tree architecture, however, as indicated by the number of primary branches, is highly variable and, therefore, not likely part of such a genetic program. Contrasting with the da neuron screen, the RP2 screen recovered no GS lines whose expression had opposite effects on growth and branching, nor lines that caused exuberant growth or branching. This could be interpreted as RP2 dendritic growth being near maximal at the developmental stage that we examined (early first instar larva). Alternatively, the lack of overgrowth phenotypes may be due to a lack of GS insertions near central dendrite overgrowth-inducing genes, or insufficient sensitivity of detection. Contrasting further with da neurons, we found that RP2 neurons generated dendritic trees of a standard size by the early first instar stage (as quantified by dendritic length and number of branches), irrespective of the territory they occupied.

Are dendrite growth and branching influenced by the same genes in peripheral and central neurons? Though both misexpression screens suggested a role for ecdysone signalling, further loss-of-function testing found that only the peripheral da neurons required the EcR. Moreover, there was remarkably little overlap (nine GS lines) between the 'growth' and 'branching' categories for peripheral da and central RP2 neurons, and no significant concordance of phenotypes generated by these nine GS lines. Only four (GSd211 (*CG7518*), GSd450 (*CG2617*), GSd454 (*CG33298*) and GSd472 (*shn*)) led to comparable growth and branching phenotypes in both types of neurons. Therefore, while growth and branching are principal features of all dendrites, central RP2 and peripheral da dendrites appear to differ significantly in the molecular pathways that regulate these features.

#### Neuronal diversity and branch order

Type-specific morphologies of dendritic trees are generated by distinct levels of growth and branching, and also by the mode of branching (for example, splitting versus interstitial), the arrangement/angles of branches (for example, acute, right or obtuse) and the types of branches (for example, spine-like protrusions versus shafts). The dendrite morphologies of the four classes of da neurons are implemented by class-specific patterns of gene expression. The simple, comb-like branching patterns of class I da neurons are governed by *abrupt *[50,51], while more complex branching patterns of class II-IV da neurons are specified by different levels of *cut*, with highest levels generating spine-like protrusions particular to class III dendrites [28]. In our da neuron screen, many GS lines exhibited class-specific effects, indicating that the unique dendrite morphologies of distinct da neuron classes can be regulated by additional factors whose activity is dependent upon the cellular context. For instance, GSd458 and GSd484 caused exuberant dendrite branching in class I neurons but the converse in class IV neurons (Table 1; Figure 2f). These findings are reminiscent of *spineless *mutants, where class I and II dendrites are increased while class III and IV arbors are reduced, with the effect that these da neurons of different classes approach a similar degree of branching complexity [29].

While it is clear that the diversity of dendrite arborisation patterns, at least among da neurons, is under genetic control, many of the underlying genetic and molecular mechanisms remain to be established. The phenotypes we recovered suggest that both the type of branches added and the order/degree of branching can be controlled separately. For example, expression of GSd422 induces spine-like protrusions, normally specific to class III da neurons, on other da classes. Branching order on the other hand is affected by four GS lines (GSd325, GSd066, GSd451, and GSd492) that cause a shift within class IV neurons from a branching morphology with higher order branches to one with lower order branches. It remains to be established whether lower and higher order branches of da neurons represent different types of dendrites, and whether phenotypes that reduce branch complexity reflect a direct effect of these GS lines on the proportion of different branch types within an arbor, the pattern of branching among dendrites of a similar type, or a partial transformation of cell identity.

Our screens provided less information about the regulation of type-specific branching patterns in central neurons, as we focused on RP2 only. While there is no evidence of different branch types among motor neuron dendrites, they do vary in the frequency of branching and length of dendritic segments: for example, higher branch orders and shorter segments are characteristic for aCC and RP2, while RP1 has lower branch orders and longer segments (M Tripodi *et al*., submitted). We identified eight GS lines (GSd017, GSd207, GSd446, GSd450, GSd462, GSd468, GSd472, GSd496), which changed the RP2 to an RP1-like dendritic morphology with relatively long segments and lower branching orders (Figure 4f–h). These observations suggest that motor neuron dendrite branching is under genetic control and that it may be linked to segment length.

#### Targeting

The territories that dendrites innervate are important determinants of neuronal function, shaping the receptive field for peripheral sensory neurons and contributing to the selection of inputs for central neurons. We did not recover dendritic targeting phenotypes in the peripheral (da) dendrite screen, yet for central (RP2) neurons these were abundant, representing more than three-quarters of all phenotypes. This may reveal fundamental differences in the way peripheral (da) and central (RP2) neurons establish their dendritic territories. It is conceivable that the formation of most da dendritic fields requires few guidance cues because their fields are: largely two-dimensional; generally explored radially; and delineated through repulsive/competitive neuro-neuronal interactions such as tiling [25,73,77,78]. Extensions of da dendrites along antero-posterior and dorso-ventral axes in the body wall may reflect responses to patterning cues or, alternatively, could result from an inherent cellular polarity. Unfortunately, no evidence to support either alternative was provided by our screen.

For central (RP2) neurons, whose dendrites are targeted to diverse regions within a complex, three-dimensional neuropile, we found abundant evidence for dendritic territories being altered by gene misexpression. This is an exciting finding since this area of dendrite development is among the least explored. Unlike in the periphery, there are as yet no indications for the existence of tiling-like mechanisms in the ventral nerve cord of *Drosophila *[30], nor restrictions of growth by neuromere boundaries (for example, GSd421; Figure 4l). It is likely, therefore, that dendritic explorations in the CNS are directed by combinations of global and local guidance cues. The large number of genes we identified as candidates provide new entry points for investigating the genetic and molecular mechanisms that underlie dendritic targeting in the CNS.

The classes of targeting phenotypes that we observed for RP2 neurons suggest that central dendrites innervate territories defined by Cartesian co-ordinates along the antero-posterior and the medio-lateral axes. The cues to establish these co-ordinates could be distributed at discrete locations within the neuropil or in the form of gradients [79] (also reviewed in [80,81]). Cues shown to be required for targeting of central neuron dendrites (for example, Sema3A, Sema1A, Slit, Netrin) are compatible with guidance along gradients [32-35,82]. In this study, we provided first evidence for the involvement of Slit and Netrin in targeting motor neuron dendrites to distinct medio-lateral neuropile territories through their receptors Robo and Frazzled. Although gradients are capable of defining a multitude of arborisation zones, relatively few zones might actually be required in the ventral nerve cord of *Drosophila*, as suggested by the actual number (five) of apparent motor neuron dendritic territories in the antero-posterior axis of each abdominal half segment [30]. Specificity of connections among the many neurons within such coarsely defined territories could be generated by additional, local cues and by functional validation of synaptic contacts.

### Common or distinct molecular mechanisms for peripheral and central dendrite morphogenesis?

The third aim of this study was to understand whether peripheral and central neurons are largely similar or different in the molecular mechanisms they employ for dendrite morphogenesis. Conservation of some mechanisms and factors required for dendrite morphogenesis have been shown to extend across neuronal cell types and phyla [7], including the cadherin Flamingo/Celsr [71,83,84] and the guidance cue Slit and its receptor Robo [33,34,82,85]. To ascertain the degree of similarity between peripheral da and central RP2 dendrite development, we determined the overlaps in the phenotypes recovered from the screens and the genes identified. Based on phenotypes, underlying cellular strategies clearly exist that are common (for example, growth and branching) as well as distinct (for example, targeting and ecdysone response). Furthermore, there was also a sizable overlap of 39% among the 62 candidate genes identified in both the da and RP2 screens, suggesting partially overlapping cytoplasmic and nuclear regulatory programs (Figure 9). However, at a more stringent level of comparison we found that particular GS lines do not necessarily induce comparable phenotypes in both cell types. In fact, candidate genes that were common to both screens were as likely to generate different phenotypes as similar ones in peripheral and central neurons. Only four genes common to both screens gave similar phenotypes in both screens (*CG7518 *(GSd211), *CG2617 *(GSd450), *CG33298 *(GSd454), and *shn *(GSd472). This observation suggests a more differentiated scenario, namely that the molecular implementation and regulation of dendrite morphogenesis in peripheral da and central RP2 neurons is largely cell type-specific, perhaps because they have distinct cellular requirements to accommodate differences in environmental complexity, the specificity of intercellular connections, or the integration of function and morphology. Nonetheless, the RP2 screen identified three genes not expressed in central neurons, but required for dendrite morphogenesis in peripheral (da) neurons (for example, *ab*, *nos*,*ttk*). We interpret this cross-fertilisation between the screens as an indication that certain cellular tasks required for the extension, branching and stabilisation of dendrites are supported by similar, though perhaps not identical, regulatory mechanisms in both cell types.

## Conclusion

We conclude that these gain-of-function screens in *Drosophila *identified new candidate genes for dendrite morphogenesis in peripheral da and central RP2 neurons. The phenotypes produced by these screens suggest that growth, branching and targeting of dendrites are regulated by pathways that are genetically separable. Direct comparison showed that 39% of the genes we identified were common to both screens, yet the phenotypes arising from the genes suggest that the dendrites of peripheral and central neurons grow, branch, and find their targets by molecular and cellular mechanisms that only partially overlap and may be largely cell type-specific. For peripheral da neurons we identified a new cell-autonomous requirement for EcR signalling during development prior to metamorphosis, when it may implement matching of dendritic territories to growing target areas. For central RP2 neurons the screens provided evidence for dendritic targeting in the neuropile, likely along Cartesian coordinates. We identified the midline signalling systems Slit/Robo and Netrin/Frazzled as candidates for dendritic targeting in the medio-lateral axis. The positioning of dendritic trees in the neuropile appeared to be independent of the developmental programs specifying patterns of growth and branching. Taken together, the identified candidate genes and phenotypes have advanced our understanding of the molecular and cellular framework within which dendrites develop.

## Methods

### Fly stocks

GAL4 driver lines were: *en-GAL4 *[21]; *Tub84B-FRT-CD2-FRT-Gal4 *[86]; *GAL4*^109(2)80 ^[15]; *GAL4*^221 ^[28]; *C161-GAL4 *[87]; and *ppk1.9-GAL4 *[17]. UAS-lines were: *UAS-mCD8::GFP *[63]; UAS-EcR-DN (*UAS-EcR-B1*^*W650A*^) [65]; UAS-IR-EcR [64]; and UAS-Dcr2 [88].

### GS expression lines

GS is a bidirectional UAS-based P-element that can activate genes on either side of the insertion site [24,89]. As described previously [23], the 141 independent GS lines screened here were pre-selected from a larger collection (1,127) for having lethal effects when misexpressed in the entire embryonic nervous system (using *scrt*^11–6^-GAL4), so as to enrich (eight-fold) for genes likely to disrupt neuronal morphology or function.

### Screening of GS lines in da neurons

Forty-four PNS neurons form per abdominal hemi-segment in three clusters [90,91]. We focused on the dorsal-most cluster in which there are eight neurons with multiple dendrites (md), including one tracheal dendrite (td) neuron, one neuron with bipolar dendrites (bd), and six dendritic arborisation (da) neurons. The pattern of dendrite outgrowth from dorsal cluster da neurons has been shown to be consistent from embryo to embryo [15,78].

For screening in embryos, each of the 141 GS lines was crossed to the recombinant fly line *GAL4*^109(2)80^, *UAS-mCD8::GFP*. Eggs were collected for 2 hours at 25°C, incubated at 25°C for another 17 hours, then shifted to 4°C for 24 hours to allow for improved visualization of the GFP signal. The resulting late stage 17 embryos were manually de-chorionated, fixed 5 minutes in 4% paraformaldehyde, immersed in halocarbon 200 oil (Halocarbon Products, River Edge, NJ, USA), coverslipped, and examined with confocal microscopy using a Yokogawa spinning disk confocal system (Perkin-Elmer, Waltham, MA, USA) on an Eclipse TE2000-U microscope (Nikon). Z-series images (60× objective) were collected using Metamorph software (MDS Inc., Mississauga, ON, Canada). Image stacks (approximately 15 optical sections, step size 0.2 μm) were exported to Photoshop software (Adobe) and prepared for publication by converting images to greyscale and adjusting brightness and contrast. Neurons from at least 20 individuals per genotype were examined.

For screening in larvae, wandering third instar larvae from crosses described above were collected just prior to pupation from uncrowded vials and dissected in 80% glycerol and 20% phosphate-buffered saline (PBS). A scalpel was used to excise the anterior end of each larva, allowing gut, fat body, and tracheal tubes to be removed. Dissected larvae were gently stretched, then squashed under a coverslip and imaged as for embryos, using a 25× objective to capture the entire dorsal cluster field or a 60× objective for detailed features of individual da neurons.

### Quantification of branching in da neurons

Maximum projections of captured confocal Z-series image stacks of ddaC neurons were compiled with Photoshop (Adobe), then imported into Reconstruct software [92] to count the number of branch ends per neuron, the length of primary branches (class III) as well as the area of each dendritic field (class IV) using the polygon method [25]. Data were exported from Reconstruct and statistical analysis was performed using Analyse-It software for Microsoft Excel. The data were tested for normal distribution using the Shapiro-Wilk test, and the probability of unequal variance was calculated with the F-test. When only one experimental condition was compared with a control, a two-tailed *t*-test was performed. For multiple experimental groups, comparisons among all pairwise combinations were made using one-way ANOVA (Tukey).

### Mosaic FLPout expression system for RP2

The *RN2-FLP *transgene was generated by modification of the e5z3 plasmid generously provided by Miki Fujioka. The lacZ coding region was replaced with a 5' linker and the Flippase coding region so as to join it to a tandem repeat of the *even-skipped (eve) *+7.9–8.6 kb regulatory element, which confers expression to the aCC and RP2 motorneurons and, weakly, the pCC interneuron [93]. Transgenic flies were generated by standard procedures. A recombinant third chromosome was generated in order to obtain a stock of the following genotype *w*^-^;; *RN2-FLP*, *Tub84B-FRT-CD2-FRT-GAL4*, *UAS-mCD8::GFP*. Thus, expression of yeast Flippase in the RP2 precursor induces recombination between the FRT sites in a stochastic fashion, inducing expression of the *UAS-mCD8::GFP *reporter under *Tub84B-GAL4 *control in isolated RP2 (and occasionally aCC and pCC) neurons.

### Screening of GS lines in RP2 neurons

Each of the 141 GS lines was crossed to the FLPout recombinant stock. Following 6 hour egg collections at 25°C, agar collection plates were transferred to 29°C for a further 20 hours. Hatched larvae were put into a drop of PBS on a microscope slide and squashed under a coverslip until flat but still intact. A minimum of five animals with several labelled RP2 neurons each were examined for each GS line under widefield fluorescence using a Zeiss Axiophot microscope, a 60×/0.9 water dipping objective (Olympus) and Zeiss AxioCam MR controlled by AxioVision 4.1 software. Reproducibility of dendritic phenotypes was confirmed for most lines by repeat crosses and confocal analysis of dissected nerve cords.

### Quantification of RP2 dendrites

For quantification ratios of anterior/posterior or medial/lateral dendrites, dendritic volumes were calculated using ImageJ [94]: first, image stacks were split into either medial and lateral dendrites (defined by the central intermediate Fasciclin II fascicle) or anterior and posterior dendrites (the midpoint being the section of axon from which the primary dendrites emerge); second, pixels showing axon and cell body were removed manually from each focal plane; third, each focal plane was converted to a binary image after removal of background by applying the same threshold to all focal planes and all image sub-stacks (for example, of anterior and posterior dendrites); fourth, the total number of pixels in the stack was then calculated as a measure of dendritic volume. These values do not represent actual but relative dendritic volumes, which allow comparisons to be carried out between parts of the same dendritic tree. Numbers of branch points, lengths of dendritic segments and total dendritic tree lengths were calculated from dendritic trees that had been reconstructed using AMIRA software (Visage Imaging, Fuerth, Germany) and a customised reconstruction module by JF Evers [42,43].

### Immunohistochemistry

Nerve cords of first instar larvae were dissected in Soerensen phosphate buffer (pH 7.4), attached dorsal side up to Poly-L-Lysine (Sigma-Aldrich, St. Louis, MO, USA) coated coverslips and fixed in 2% paraformaldehyde, 3% sucrose in PBS for 60 minutes at room temperature. Washes were done in PBS with 0.3% Triton-X-100 (Sigma), 5 changes over the course of 60 minutes. Embryos and third instar larvae were dissected in PBS, fixed for 10 minutes in 4% paraformaldehyde, then washed for 2 hours at room temperature. Specimens were incubated in primary antibodies overnight at 4°C, washed, incubated in secondary antibodies either for 2 hours at room temperature or overnight at 4°C. In some cases, the muscles overlying the dorsal cluster PNS neurons were removed for better visualization of da neurons. Specimens were mounted in Vectashield (Vector Laboratories, Burlingame, CA, USA) and imaged with a SP1 point scanning confocal system (Leica) or a CSU-22 spinning disk confocal system (Yokagawa), operated with MetaMorph (Molecular Devices).

Primary antibodies were: anti-Fasciclin II (MAb 1D4) supplied by the Developmental Studies Hybridoma Bank (DSHB; 1:20 dilution), goat anti-GFP (1:1,500; Abcam, Cambridge, MA, USA) and mouse anti-EcR antibodies (1:10 dilution; DSHB). The mouse anti-EcR antibodies included: all EcR isoforms (mAb Ag10.2); EcR-A (mAb 15G1a); or EcR-B1 (mAb AD4.4). Primary antibodies were diluted in PBS, 0.3% Triton-X-100. Secondary antibodies were Alexa fluorophore (Alexa488, Alexa561 and Alexa633; Invitrogen, Carlsbad, CA, USA) and Rhodamine Red-X conjugated secondaries (Jackson ImmunoResearch, West Grove, PA, USA), highly cross-absorbed applied at a 1:800 dilution in PBS, 0.3% Triton-X-100.

### *In situ *hybridisation

*In situ *hybridization was performed as described in [95] with 0.3% SDS present in the hybridisation solution. Probes were generated from cDNA clones (*Drosophila *Genomics Resource Center) using the Megascript kit (Ambion/Applied Biosystems, Austin, TX, USA) and DIG-UTP (Roche, Basel, Switzerland) with the following primers. pOT2: 5', ATTAACCCTCACTAAAGGGAGCAGATCTGATATCATCGCCACT; 3', TAATACGACTCACTATAGGGAGAACGCGGCTACAATTAATACATAACC. pBSK^-^: 5', GCGCGCAATTAACCCTCACTAAAGGG; 3', GCGCGCGTAATACGACTCACTATAGGG. pOTB7: 5', ATTAACCCTCACTAAAGGGACTAAGGTAGCGAGGCCTGGGTGG; 3', GCGCGCAATTAACCCTCACTAAAGGG.

### MARCM analysis to study the cell-autonomous effects of *usp *mutations in da neurons

To characterize the mutant phenotype of *usp *in da neurons, flies of the stock *FRT19A*, *tub-GAL80*, *hs-FLP*; *GAL4*^109(2)80^, *UAS-mCD8::GFP *were crossed to flies from the following stocks: 1. *FRT19A*, *w*^+ ^2. *FRT19A*, *UAS-mCD8::GFP*, *usp*^2^; λ10,Tb/TM3 3. *FRT19A*, *usp*^3^/*FM7c *and 4. *FRT19A*, *usp*^5^/*FM7c*. Embryos were collected for 2 hours, incubated at 25°C for 2–3 hours, then heat-shocked at 38°C for 1 hour and incubated at 25°C until they were analyzed as wandering third instar larvae, just prior to pupation.

## Abbreviations

AEL: after egg laying; CNS: central nervous system; da, dendritic arborisation; EcR: ecdysone receptor; GFP: green fluorescent protein; GS: Gene Search; PBS: phosphate-buffered saline; PNS: peripheral nervous system; RNAi: RNA interference; Usp: Ultraspiracle.

## Competing interests

The authors declare that they have no competing interests.

## Authors' contributions

YO and BC contributed equally; ML and DvM contributed equally.YO performed all experiments related to the study of da neurons. BC performed all experiments related to the study of RP2 neurons. ML designed the FLPout strategy for RP2 neurons. ML and DvM conceived of the study, participated in its design and coordination and drafted the manuscript. All authors read and approved the final manuscript.

## Supplementary Material

Additional file 1 Summary of da and RP2 dendrite screens. Summary of da and RP2 dendrite screens. Click here for file

Additional file 2 Extent of dendritic branching in the antero-posterior axis. The extent (distance in μm) spanned by dendritic trees in the antero-posterior axis was measured for control RP2 neurons at 25–31 hours AEL and GS lines GSd466, GSd312, GSd446 and GSd450. Dendritic growth and numbers of branch points are reduced by expression of every one of these GS lines (Figure 4f). However, 'growth' and 'branching' phenotypes differ in that 'growth' (GSd466, GSd312) but not 'branching' (GSd446, GSd450) phenotypes show a clearly reduced dendritic exploration of the neuropile (for example, dendritic extent) in the antero-posterior axis. *P < 0.01, **P < 0.005, *t*-test, N = 5, error bars indicate the standard error. Click here for file

Additional file 3 Expression patterns of candidate genes identified in the RP2 screen. Expression patterns are shown as revealed by whole mount *in situ *hybridisation. Panels are subdivided into two groups, those exhibiting expression in subsets of cells and those with ubiquitous expression throughout the CNS. Anterior is left and a ventral view of the ventral nerve cord is shown. Scale bar: 50 μm for all panels, except *CG14709 *where it represents 25 μm. Click here for file

Additional file 4 Misexpression phenotypes implicating ecdysone signalling in dendrite morphogenesis. **(a) **Dendrites of dorsal cluster da neurons in control third instar larva *(GAL4*^109(2)80^, *UAS-mCD8::GFP/+*). **(b-d) **Arborisation defects observed in larvae misexpressing GSd113 (*Kr-h1*), GSd332 (*bon*), or GSd327 (*Hr38*). **(e) **Control RP2 neurons at 25–31 hours AEL, visualized by wide-field fluorescence microscopy. **(f-h) **Effects of RP2 misexpression of GSd204 (*Kr-h1*), GSd332 (*bon*), or GSd327 (*Hr38*). Scale bars: (a-d) = 100 μm; (e-h) = 10 μm. Click here for file

Additional file 5 Expression of EcR isoforms in all dorsal cluster da neurons. Dorsal cluster da neurons of embryos (stage 16–17) or late third instar larvae (genotype: *GAL4*^109(2)80^, *UAS-mCD8::GFP*) labelled for GFP and one of three monoclonal antibodies that detect either: all EcR isoforms (mAb Ag10.2); EcR-A (mAb 15G1a); or EcR-B1 (mAb AD4.4). Each EcR antibody labels the nuclei of all six da neurons of the dorsal cluster, in addition to the tracheal dendrite neuron and bipolar dendrite (bd) neuron, which are also detected by *GAL4*^109(2)80^. Nearby, additional nuclei, including the large epidermal cell nuclei of third instar larvae, are also labelled by EcR antibodies and shown in these maximal Z-projections of stacked confocal images. Anterior is left and ventral is down. Click here for file
