# Supplementary material for: Identification of genes influencing dendrite morphogenesis in developing peripheral sensory and central motor neurons
Source: Neural Dev. 2008 Jul 10;3:16. doi: 10.1186/1749-8104-3-16 (PMC2503983; doi:10.1186/1749-8104-3-16)
Supplement: Additional file 1 — Summary of da and RP2 dendrite screens. Summary of da and RP2 dendrite screens. [file 1749-8104-3-16-S1.doc]

| **Summary of da and RP2 Dendrite Screens** | | |
| --- | --- | --- |
|  | **da** | **RP2** |
| Number of GS lines screened | 141 | 141 |
| Number of GS lines causing phenotypes | 43 | 60 |
| Number of GS lines causing Growth phenotypes | 17 | 11 |
| Number of GS lines causing Branching phenotypes | 39 | 9 |
| Number of GS lines causing Targeting phenotypes | 0 | 52 |
| Number of genes with more than 1 GS insertion | 6 | 13 |
| Number of GS lines with insertions in unique loci | 34 | 47 |
| Number of genes near sites of unique GS insertions | 35 | 51 |
