# Supplementary figures and images for: Identification of genes influencing dendrite morphogenesis in developing peripheral sensory and central motor neurons
Source: Neural Dev. 2008 Jul 10;3:16. doi: 10.1186/1749-8104-3-16 (PMC2503983; doi:10.1186/1749-8104-3-16)

# Maximal anterior-posterior extent of dendrites (in $\mu\text{m}$ )

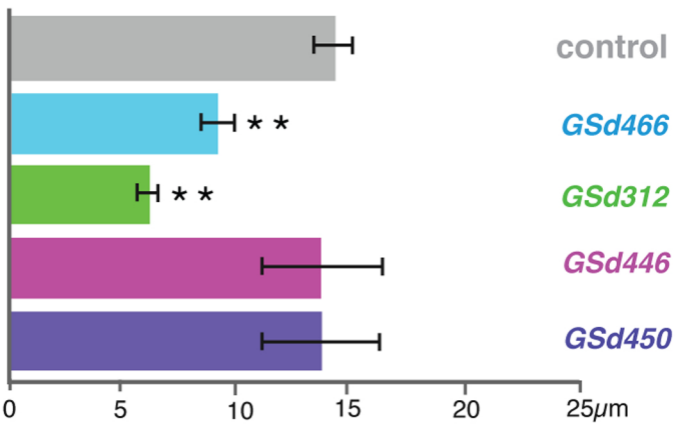

Supplement: Additional file 2 — Extent of dendritic branching in the antero-posterior axis. The extent (distance in μm) spanned by dendritic trees in the antero-posterior axis was measured for control RP2 neurons at 25–31 hours AEL and GS lines GSd466, GSd312, GSd446 and GSd450. Dendritic growth and numbers of branch points are reduced by expression of every one of these GS lines (Figure 4f). However, 'growth' and 'branching' phenotypes differ in that 'growth' (GSd466, GSd312) but not 'branching' (GSd446, GSd450) phenotypes show a clearly reduced dendritic exploration of the neuropile (for example, dendritic extent) in the antero-posterior axis. *P < 0.01, **P < 0.005, t-test, N = 5, error bars indicate the standard error. [file 1749-8104-3-16-S2.pdf]

## expression in subsets of cells

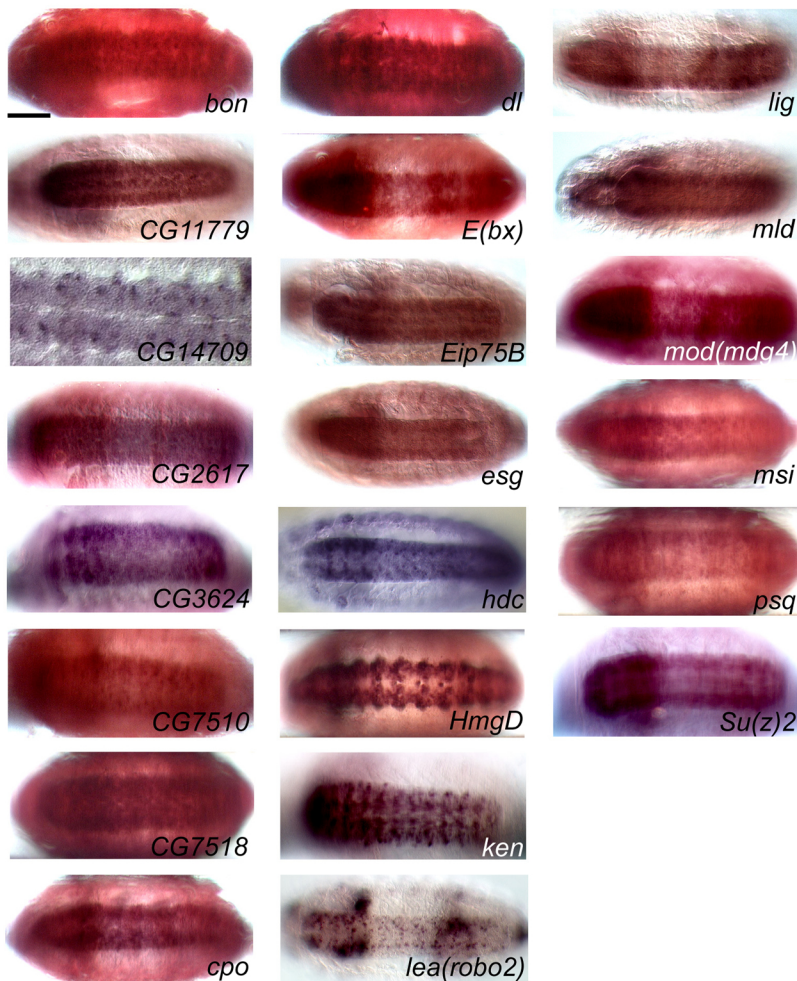

## ubiquitous expression

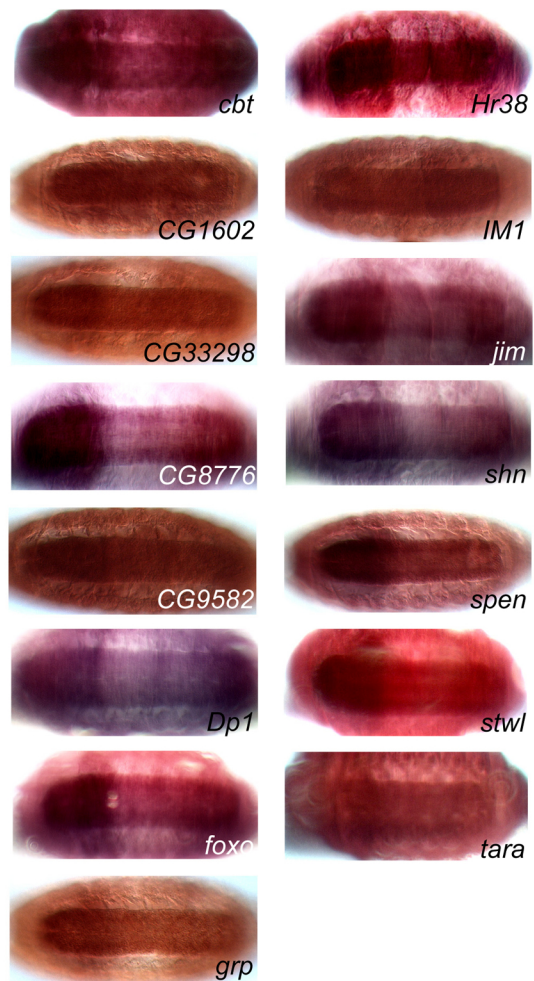

Supplement: Additional file 3 — Expression patterns of candidate genes identified in the RP2 screen. Expression patterns are shown as revealed by whole mount in situ hybridisation. Panels are subdivided into two groups, those exhibiting expression in subsets of cells and those with ubiquitous expression throughout the CNS. Anterior is left and a ventral view of the ventral nerve cord is shown. Scale bar: 50 μm for all panels, except CG14709 where it represents 25 μm. [file 1749-8104-3-16-S3.pdf]

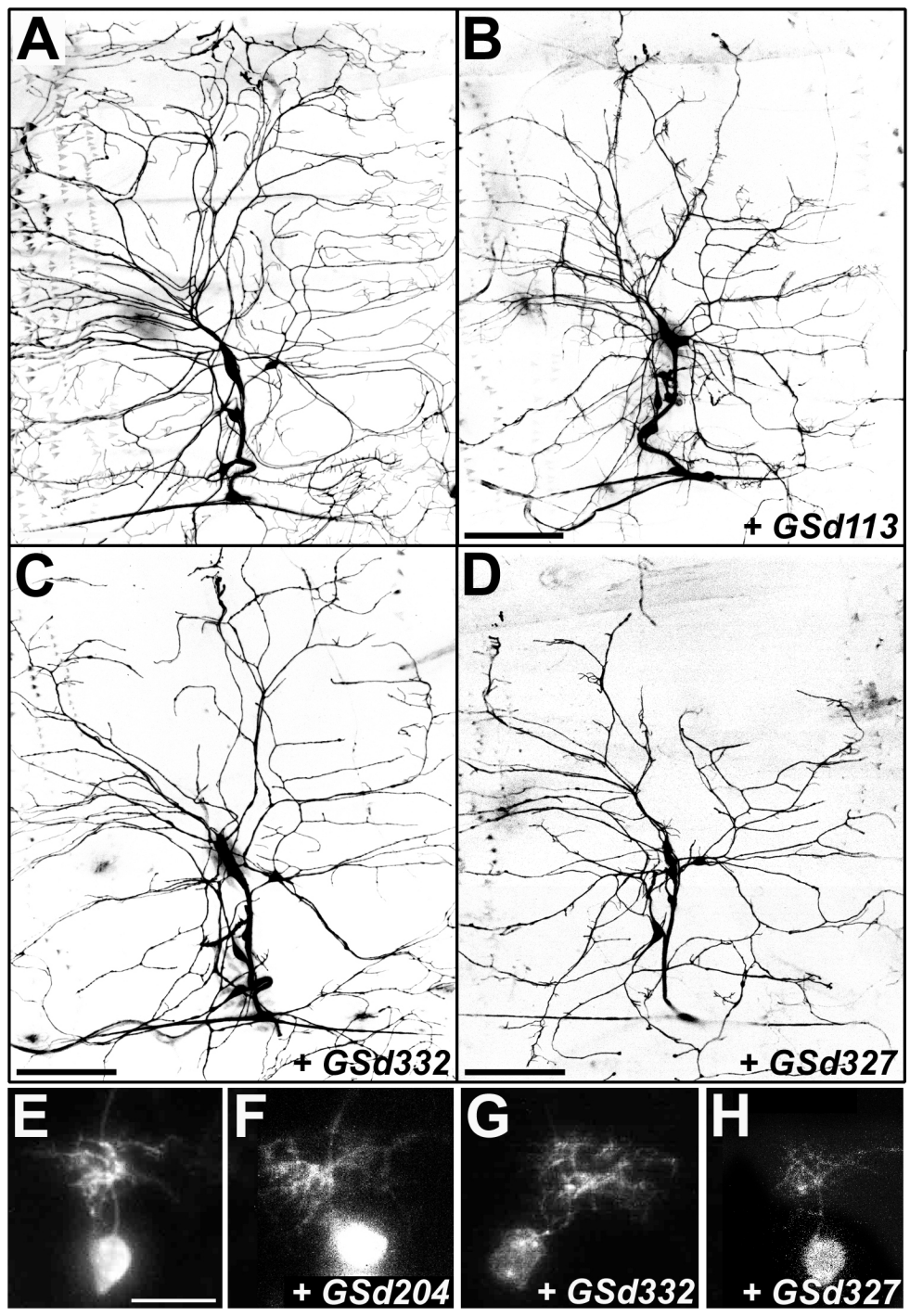

Supplement: Additional file 4 — Misexpression phenotypes implicating ecdysone signalling in dendrite morphogenesis. (a) Dendrites of dorsal cluster da neurons in control third instar larva (GAL4109(2)80, UAS-mCD8::GFP/+). (b-d) Arborisation defects observed in larvae misexpressing GSd113 (Kr-h1), GSd332 (bon), or GSd327 (Hr38). (e) Control RP2 neurons at 25–31 hours AEL, visualized by wide-field fluorescence microscopy. (f-h) Effects of RP2 misexpression of GSd204 (Kr-h1), GSd332 (bon), or GSd327 (Hr38). Scale bars: (a-d) = 100 μm; (e-h) = 10 μm. [file 1749-8104-3-16-S4.pdf]

**Embryo (st.16-17)**

**Larva (late L3)**

**EcR**

**GFP**

**Overlay**

**EcR**

**GFP**

**Overlay**

**EcR(all)**

**EcR-A**

**EcR-B1**

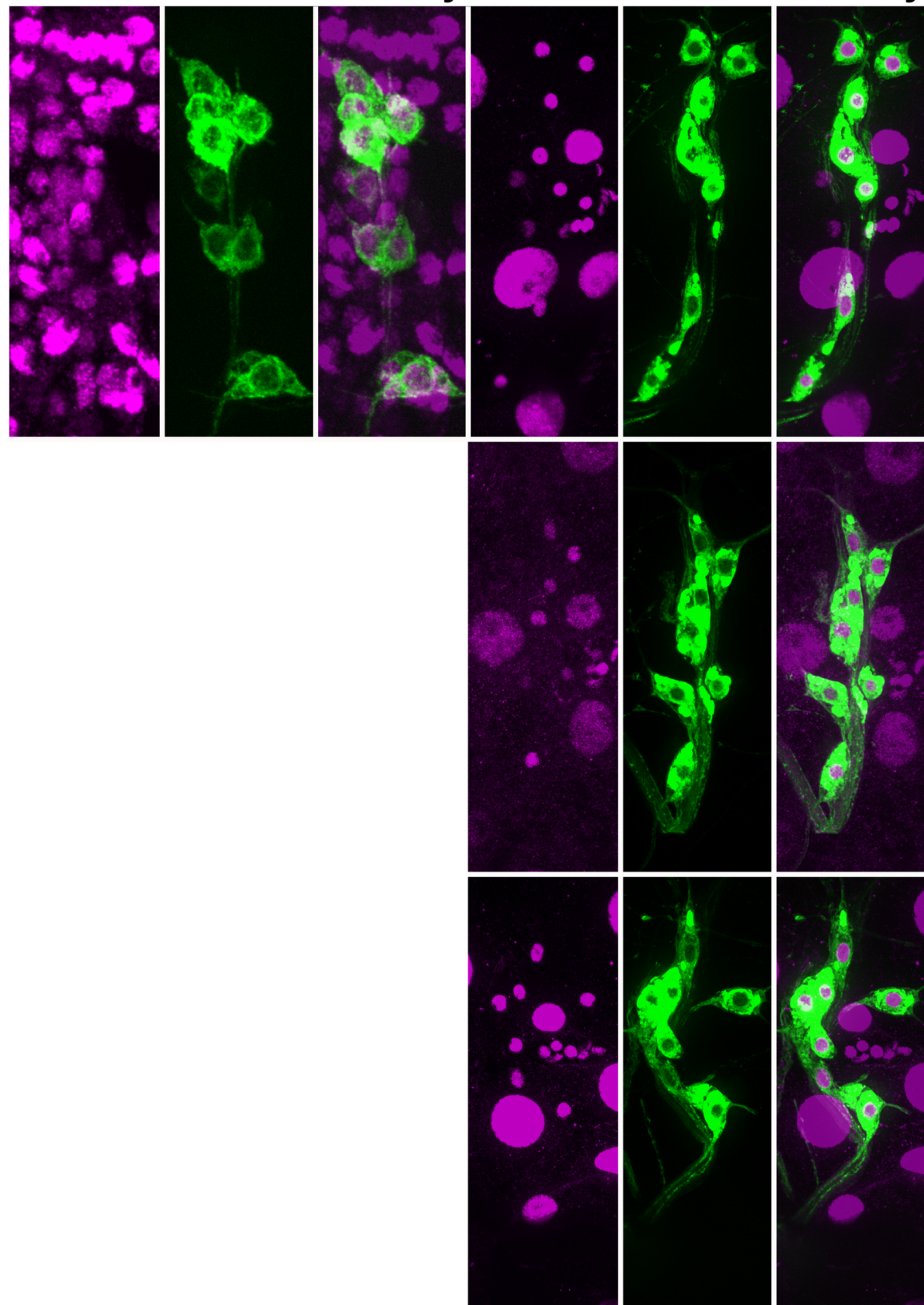

Supplement: Additional file 5 — Expression of EcR isoforms in all dorsal cluster da neurons. Dorsal cluster da neurons of embryos (stage 16–17) or late third instar larvae (genotype: GAL4109(2)80, UAS-mCD8::GFP) labelled for GFP and one of three monoclonal antibodies that detect either: all EcR isoforms (mAb Ag10.2); EcR-A (mAb 15G1a); or EcR-B1 (mAb AD4.4). Each EcR antibody labels the nuclei of all six da neurons of the dorsal cluster, in addition to the tracheal dendrite neuron and bipolar dendrite (bd) neuron, which are also detected by GAL4109(2)80. Nearby, additional nuclei, including the large epidermal cell nuclei of third instar larvae, are also labelled by EcR antibodies and shown in these maximal Z-projections of stacked confocal images. Anterior is left and ventral is down. [file 1749-8104-3-16-S5.pdf]
